# Elastomeric tubes with self-regulated distension

**DOI:** 10.1016/j.isci.2022.104369

**Published:** 2022-05-06

**Authors:** Nathan Jen, Guilherme M. Bessa, Kaelyn Nicolson, Jianliang Xiao, David S. Nobes, Hyun-Joong Chung

**Affiliations:** 1 Department of Chemical and Materials Engineering, University of Alberta, Edmonton, AB, Canada; 2 Department of Mechanical Engineering, University of Alberta, Edmonton, AB, Canada; 3 Department of Mechanical Engineering, University of Colorado Boulder, Boulder, CO, United States

**Keywords:** Robotics, Polymers, Materials mechanics

## Abstract

Compliant elastomer tubing with a fabric “jacket” has been essential in various applications as soft robotic actuators, such as in biomedical exomuscles and massage therapy implements. Here, our study shows that a similar design concept can be an effective strategy in realizing passive regulation in the tube’s distension, as well as in preventing aneurysm-like asymmetric rupture of the tube. A custom hydraulic pressure testing rig was built to perform experiments. The jacketed tubes initially deform rapidly as pressure increases, but a self-regulation behavior suppresses the tube’s continued distension by strain-stiffening of the “jacket”. In addition, highly asymmetric distension, common to elastomeric tubes due to imperfection in fabrication, is prevented dramatically by the “jacket”. A three-dimensional finite element model predicts the distension of all tested tubes quantitatively across the entire experimental pressure ranges and beyond. Incorporating custom-designed kirigami relief patterns in the “jackets” expands the potential of the elastomeric tubes.

## Highlights

• Fabric “jackets” prevent asymmetric “aneurysms” in pressurized elastomeric tubes
• Using FEM, we precisely modeled distension of pressurized, jacketed elastomer tubes
• Hysteresis must be considered in predicting the deformation of elastomeric tubes
• Fabric “jackets” may have kirigami relief patterns to realize complex deformations

## Graphical abstract

**Figure:**
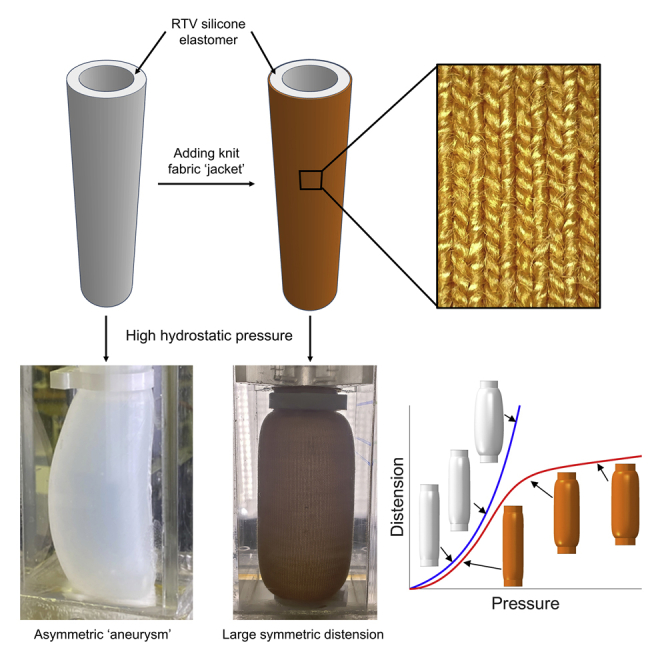

## Introduction

From robotics, to industry, to medical devices, development of system components which can accurately respond to and control a flowing fluid has become an incredibly important endeavor. Components which can passively respond to fluid behavior due to their inherent material properties are specifically valuable, as design of an integrated system to actively monitor and respond to changing fluid flow can be very expensive from both a logistical and financial standpoint.

Use of bare elastomers to passively regulate flow in microfluidic channels has been previously reported in literature for many decades (Chappel, 2020). This is generally accomplished by designing a channel with elastomeric walls which contract as pressure increases, regulating the flow rate (or restricting it entirely if pressure is sufficiently high) although more complicated designs have been reported (Mosadegh et al., 2010). Applications range widely, from drug delivery to agriculture to lab-on-a-chip devices (Zhang et al., 2020; Zhangzhong et al., 2013; Doh and Cho, 2009). However, these devices are generally on the scale of tens of micrometers, where the prevailing fluid forces are viscous rather than volumetric.

Larger-scale elastomeric channels with strictly regulated distension behavior must accommodate for different fluid flow properties as well as greatly increased volumetric forces—especially so in hydraulic instances, where the fluid is incompressible and its density may easily be hundreds of times greater than commonly used gases. One such instance is the *ex vivo* heart perfusion device (EVHP), a medical device which keeps a donor heart alive *ex vivo* by connecting it to a tubing system and pumping a blood substitute through it (White et al., 2013). The tubing immediately connected to the donor organ in this device is on the centimeter scale and should ideally be compliant enough to act as a shock absorber for the pulsatile fluid flow (which occurs at pressures in the tens of kPa) without being so compliant as to rupture during operation (Zhalmuratova et al., 2019). This replicates the function of human aorta, which displays this behavior *in vivo* and displays a distinct “J-shaped” stress-strain curve when tested under uniaxial tension.

Zhalmuratova et al. (Zhalmuratova et al., 2019) suggested the use of fiber-elastomer composites as material for this compliant tubing. Fiber-elastomer composites, which consist of stiff fibers embedded within a compliant elastomeric matrix, have garnered much attention in the scientific community for their properties which combine the most desirable aspects of both component materials. Specifically, the elastomeric matrix provides a robust and deformable base for the material which allows it to withstand many different stresses, while the stiffer fibers act as a reinforcement to prevent excessive deformation under large stress magnitudes. Under uniaxial tension, the same “J-shaped” stress-strain curve seen in aorta and many other biological tissues is also observed. Preferential or prescribed orientation of the fibers leads to the creation of anisotropic materials, which display resistance to certain deformation modes (O’Connor, 1977; Beter et al., 2020; Chatterjee et al., 2021).

**Figure 1:**
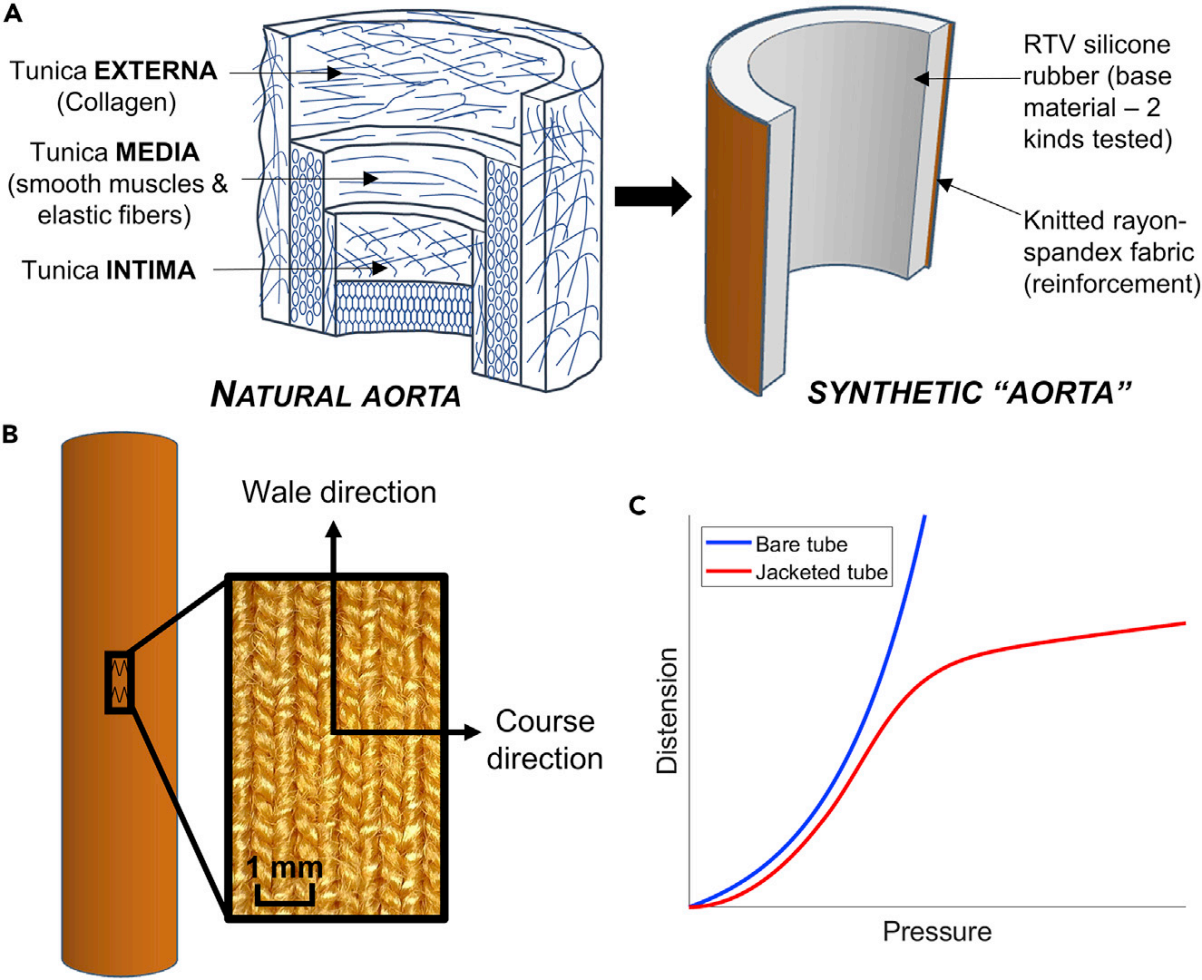
Overall design concepts (A) Schematic showing the initial biomimetic inspiration for the design of the fabric-jacketed elastomeric tubes. (B) Schematic illustrating the knit directions of the fabric and their orientations relative to the tube. (C) Example plot showing trends in radial distension vs. pressure for jacketed and unjacketed tubes under hydrostatic pressure.

The versatility of fiber-elastomer composites enables their use in soft robotic actuators (Marchese et al., 2015; Wang et al., 2021; Hubbard et al., 2019; Singh and Krishnan, 2020; Feng et al., 2020; Buffinton et al., 2020), biomimetic, or biomedical devices (Zhalmuratova and Chung, 2020; Ramakrishna et al., 1987; Bailly et al., 2014), flexible yet tear- or impact-resistant garments (King et al., 2015), devices found in harsh tribological settings such as tire treads (Jung and Sodano, 2020; Khafidh et al., 2019), and even heat-shielding layers for space vehicles (George et al., 2018). In the context of tubing for an EVHP device, the soft elastomeric matrix of the composite dominates much of the material response when pressurized while an embedded fabric layer facilitates strain-stiffening if the tubing becomes overpressurized, preventing rupture. In this way, the material could successfully act as a “mock aorta”, replicating both the structure (Figure 1A) and the function of biological aorta (which itself is a fiber-reinforced material exhibiting J-shaped stress-strain behavior) in regulating somatic blood flow via the so-called Windkessel effect (Belz, 1995). Furthermore, such tubing is hypothesized to have great utility as a general-purpose macroscale elastomeric flow regulator or as a static actuator with self-regulating distension in the radial direction.

In our development of tubular structures, the “embedded fibers” design later gave way to a simplified alternative in which the fabric layer was not embedded, and instead wrapped around the tubing as a “jacket” (Figure 1A). This was hypothesized to retain most of the biomimetic behaviors sought after in the embedded model, while also allowing for greater movement of fibers and being easier to manufacture. While the term “fiber-elastomer composite” generally refers to materials with embedded fibers, this alternative design is not without precedent. In fact, a spectrum of attachment methods for hollow or tubular complexes of fibrous and elastomeric materials has been reported in literature. These methods include cast elastomer-based structures with fully embedded fibrous layers (Martinez et al., 2012; Galloway et al., 2013; Chen et al., 2021), fiber-based structures with elastomeric layers that are laminated or otherwise adhered together (Mandlekar et al., 2022; Nguyen and Zhang, 2020; Clapp et al., 2016), braided fiber-based structures which are impregnated with elastomeric resin (Ayranci and Carey, 2008), and “jacketed” elastomeric structures with one or more external layers of fibrous reinforcement that are not specifically adhered to the surface (Sangian et al., 2015; Tiwari et al., 2012; Connolly et al., 2015; Sridar et al., 2016; Belforte et al., 2014; Natividad and Yeow, 2016; Simpson et al., 2017; Zhu et al., 2020; Zhang et al., 2019). The lattermost design is further explored to provide additional context for this work.

The most well-known application of jacketed elastomeric tubing is the McKibben actuator, which sees use in various soft robotic applications. First developed in 1958 (Tondu, 2012), this device consists of an elastomeric tube surrounded by a braided layer of fabric, which moves longitudinally when pressurized pneumatically or hydraulically due to the contraction of the surrounding fibers (Sangian et al., 2015; Tiwari et al., 2012). In the 60+ years since, countless variations on this base design have been published. For example, Connolly et al. (2015) expanded upon this concept by attaching several such actuators in series with different patterns in the surrounding fibers to produce complex actuation modes. Also, Sridar et al. (2016) used extremely stiff surrounding fabric to produce an actuator that extended longitudinally and stiffened radially while pressurized. Fairfield proposed that hydraulic McKibben actuators can be useful in an irrigation soft robot, although a physical prototype of this design has not been built to the best of our knowledge (Fairfield, 2020).

In the biomedical field, Belforte et al. (2014) created many McKibben actuator-inspired prototypes of similar fabric-reinforced soft actuators for various biomedical purposes, such as massage therapy for patients suffering from lymph edema. Roche et al. (2014) used McKibben actuators completely embedded in an elastomeric matrix to facilitate complex deformation modes in a synthetic model of the human left ventricle. Natividad et al. (Natividad and Yeow, 2016) used an elastomeric “bladder” surrounded by a heat-sealed fabric jacket and placed on the brachium as an assistive device for shoulder abduction for people with cerebral palsy. Simpson et al. (Simpson et al., 2017) used a similar design with a sewn sleeve to act as an “exomuscle” to help facilitate arm movement in stroke victims. Finally, Zhu et al. (2020) developed flexible muscle “sheets” which contained prescribed arrays of elastomeric tubing within a sewn fabric “shell”; these sheets were capable of several actuation modes depending on the tubing patterns, including gripping, bending, and twisting.

Despite these numerous applications, fewer attempts have been made to fully explain, model, and predict the distension behavior of jacketed elastomeric tubing at a base level. Doing so successfully requires taking into account a combination of several key material properties which must all be considered in modeling the material response:

• **Hyperelasticity**: Elastomers are a classic example of a hyperelastic material, that is, one whose deformation behavior is nonlinear and governed by a strain energy density function rather than a constant factor. (Valanis and Landel, 1967) Many constitutive models exist to describe the behavior of different hyperelastic materials, and there is no universal agreement in literature on which should be used. Furthermore, for any given hyperelastic material, there may be great disagreement between sources on the material coefficients, even when the same constitutive model is used (Xavier et al., 2021).
• **Strain-stiffening**: When subject to a uniaxial tensile test, elastomers display distinct strain-stiffening behavior at large strains (in the hundreds of percents). Addition of embedded fibers will greatly accelerate the onset of this effect (Zhalmuratova et al., 2019). It is hypothesized that even if the fibers form a surrounding layer (such as a fabric jacket) rather than being embedded, the same strain-stiffening effect occurs.
• **Anisotropy**: Addition of a knit fabric, which has a repeating, directional structure (Figure 1B) induces anisotropy in the structure. The stiffness of the tube will have a directional dependence (highest parallel to the directions of fiber elongation), which requires complicated constitutive models to properly explain (Holzapfel et al., 2000).
• **Hysteresis**: Elastomers, like all rubbers, are subject to material phenomena that increase their compliance after repeated loading-unloading cycles. The most significant of these is the Mullins effect (Diani et al., 2009), a complicated and multi-faceted phenomenon that results in gradual and irreversible increases in compliance over many strain cycles. Fabrics, including knit fabrics, have been observed to display the same hysteresis behavior as well when under cyclic stress (Cooper et al., 1965; Matsuo and Yamada, 2009).

Some works opt to forgo discussing these complicating factors entirely and focus solely on the behavior of their devices with respect to their intended applications (e.g. displacement or bending angle for a soft robotic actuator). Others provide some form of modeling, but use it primarily as a way to qualitatively validate the trends in their experimental results. The parallel pipe-crawling soft robot designed by Zhang et al. (Zhang et al., 2019) is an example of a work on jacketed elastomeric tubing in which the deformation behavior is deeply studied and preemptive numerical modeling is actually used to optimize the fabrication parameters of the prototype, although hysteresis behavior after repeated usage is not investigated. It is also notable that the vast majority of jacketed elastomeric tubing devices in published literature operate on the scale of a few millimeters, much smaller than the tubing investigated in this work.

We present a highly tunable design for centimeter-scale elastomeric tubing wrapped with a knit fabric jacket; changing the material composition, dimensions, or construction of the jacket can greatly alter the self-regulation response. Tubes were tested under hydrostatic pressure on a custom-built hydraulic flow loop with ends clamped in place, and trends in radial distension were measured. The jacketed tubing is initially very compliant when first pressurized, but displays marked self-regulation behavior at high pressures that is not present in bare elastomeric tubes (Figure 1C). In addition, it was found that the subtle softening of the tube material due to stretch/release cycle has tremendous effect in the degree of distension. 3D finite element simulations were created using material coefficients obtained from uniaxial, quasi-hysteresis tensile tests of the materials of interest, and were observed to closely replicate the experimental distension trends while also predicting a continuation of the observed trends at pressures beyond the experimental range.

## Results

### Hydrostatic pressure testing results

Elastomeric tubes were cast in custom 3D printed molds using two commercially available silicone rubbers: Ecoflex 00-50 (EF) and Dragon Skin 10 SLOW (DS). Fabric jackets were cut from a knit fabric blend of 93%–7% rayon-spandex and wrapped around the fabric such that the wale direction ran parallel to the longitudinal axis of the tube (Figure 1B).

**Figure 2:**
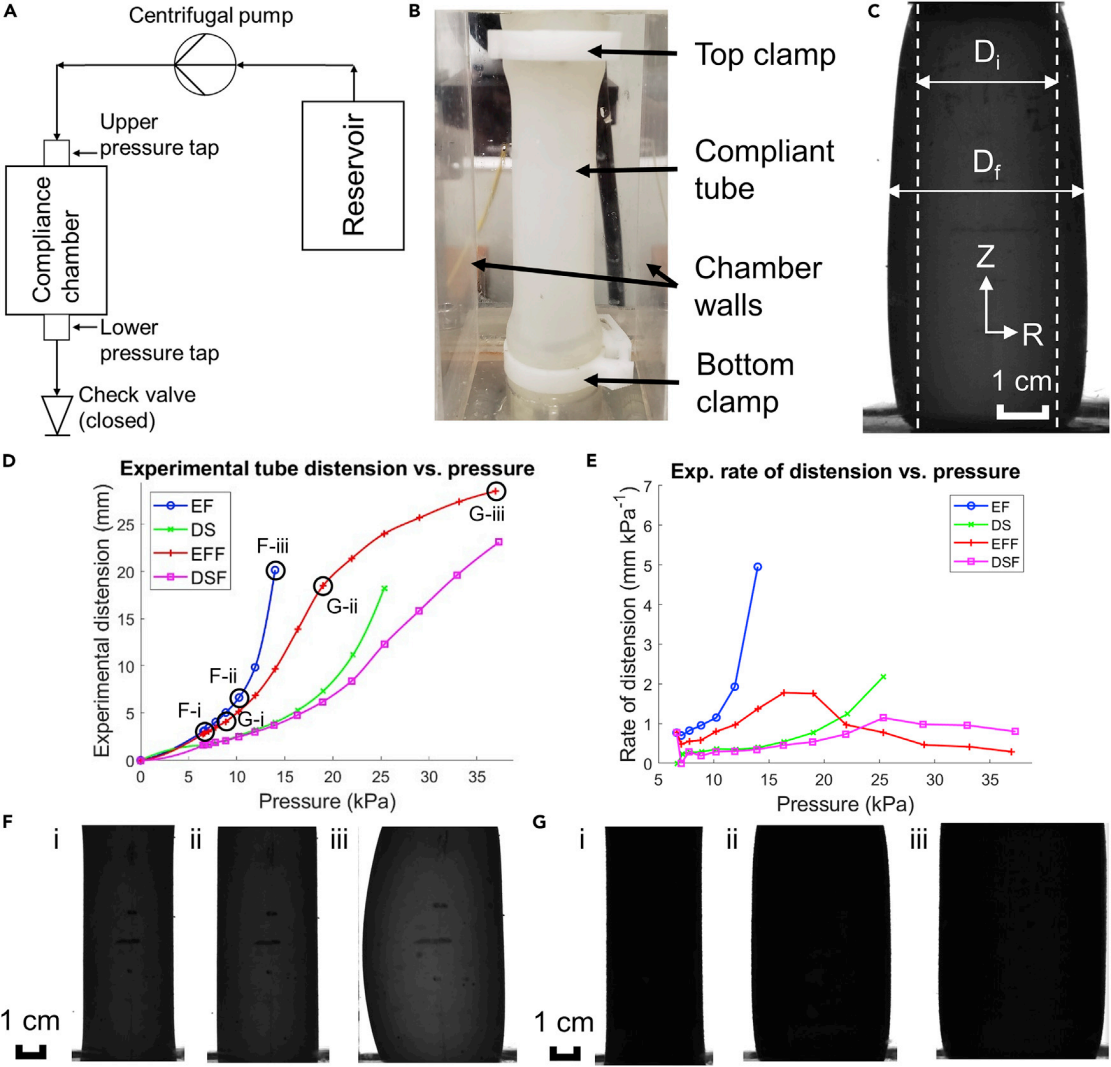
Experiments on tubes experiencing hydraulic internal pressure For a Figure360 author presentation of this figure, see https://doi.org/10.1016/j.isci.2022.104369. (A) Schematic drawing of the pressure testing system used. (B) Closeup of the compliant chamber with elastomeric tube inserted. (C) Image of distended elastomeric tube, with initial and final diameter measurements shown. Scale bar: 1 cm. (D) Plot of radial tube distension (mm) vs. pressure (kPa); EF = Ecoflex, DS = Dragon SKin, EFF = Ecoflex + fabric, DSF = Dragon Skin + fabric. (E) Plot of rate of distension (mm kPa^−1^) vs. pressure (kPa). (F) Experimental images for bare Ecoflex tubes at pressures of (i) 6.52 kPa, (ii) 10.25 kPa, and (iii) 13.99 kPa. Scale bar: 1 cm. (G) Experimental images for jacketed Ecoflex tubes at (i) 8.84 kPa, (ii) 18.99 kPa, and (iii) 36.95 kPa. Scale bar: 1 cm. See also Figures S1–S4.

Bare (unjacketed) and jacketed EF and DS tubes (denoted EFF and DSF respectively) underwent hydrostatic pressure testing in a custom-built hydraulic “flow loop” (schematic shown in Figure 2A). During testing, a single tube was clamped in place in the compliant chamber (Figure 2B) and increasingly pressurized by running the centrifugal pump at incrementally faster speeds. Tests continued until significantly asymmetric distension of the tube was observed such as in Figure 2F:iii.

Tested tubes were held at each pressure level until the distension stabilized, approximately 2–3 min, to allow for any relaxation of the material. Then, a camera facing the compliant chamber took images of the tubes at each pressure level (Figure 2C), which were later analyzed to measure the extent of distension. “Distension”, in this context, is defined as the difference between the initial and final diameters (Figure 2C) where final diameter is taken as an average value over the middle 3 cm of the image. It is represented as the variable δ (mm) in Equation 1 below:

(Equation 1) $$\delta =\frac{1}{j_{max}}\sum_{j=1}^{j_{max}}\left(D_{f,j}-D_{i}\right)$$

where the range [1, $j_{max}$] represents the number of measurements taken in the middle 3 cm of the image (also corresponding to the number of pixels in 3 cm of the image), $D_{f,j}$ is the final outer diameter measurement at point *j* (mm), and $D_{i}$ is the initial outer diameter of the tube (mm). $D_{i}$ has a constant value of 27.05 mm for unjacketed tubes and 28.21 mm for jacketed tubes.

A plot of distension vs. pressure values for all tested tubes is shown in Figure 2D and some corresponding real-life images are shown in Figures 2F–2G. The hydrostatic pressure test for the jacketed tubes could not be carried out until severe asymmetric distension occurred due to the limitations of the flow loop system. Plots of all experimental results can be found in the supplemental information, Figures S1–S4.

The most notable trend is the difference in the shape of the curves for unjacketed and jacketed tubes. Unjacketed tubes display an exponential growth curve, rapidly approaching failure with increasing pressure. Conversely, jacketed tubes display a sigmoidal curve in which the exponential growth begins but is eventually halted, entering a region of self-regulation in which the increasing tube distension slows and even appears to plateau.

Figure 2E illustrates the self-regulating effect experienced by the jacketed tube at the highest pressures. The abscissa is pressure at each data point as shown in Figure 2D, whereas the ordinate is the “rate of distension”, that is, the unit increase in distension per unit increase in kPa between each point and the one directly previous. While the rate of distension only increases across the pressure range for EF and DS tubes, it peaks and eventually decreases for the jacketed tubes.

The other main result is related to the symmetry of distension observed at high pressures in Figures 2F–2G. While the EF tube experiences a sudden onset of asymmetric distension near the end of its pressure range (Figure 2F:iii), the EFF tube maintains a relatively symmetric distension profile even at pressure over twice the maximum experienced by the EF (Figure 2G:iii). Plots quantifying this asymmetry are found in the supplemental information, Figures S5, S6, S7, and S8.

### Uniaxial tensile testing results

ABAQUS requires that the coefficients of an appropriate constitutive model, selected from a list, are associated with the materials being simulated. Therefore, there were two important decisions to make: 1) the constitutive material model selected to represent the behavior of both the elastomers and fabric used, and then 2) the material coefficients for each. Further background information on the equations used in this process is found in the supplemental information.

The Mooney-Rivlin model was selected for the elastomers based on its established accuracy in describing deformation behavior of silicone elastomers (Zhalmuratova et al., 2019). For a uniaxial tensile test, the Mooney-Rivlin model takes the form of Equation (2):

(Equation 2) $$t_{1,elastomer}=2C_{10}\left(\lambda_{1}^{2}-\frac{1}{\lambda_{1}}\right)+2C_{01}\left(\lambda_{1}-\frac{1}{\lambda_{1}^{2}}\right)$$

where $t_{1,elastomer}$ is the true stress in the direction of uniaxial elongation (Pa), $\lambda_{1}$ is the stretch ratio in the direction of elongation (mm mm^−1^), and $C_{10}$ and $C_{01}$ (both Pa) are the coefficients of interest that must be fit to the uniaxial tensile test data.

For the fabric, the Holzapfel-Gasser-Ogden (HGO) model was selected as it was originally conceived to describe the hyperelastic anisotropic behavior of aortic tissue, one of the original inspirations for the fabric-jacketed tube design (Holzapfel et al., 2000). Ordinarily, the HGO model takes the form shown in Equation (3):

(Equation 3) $$t_{1,HGO}=2C_{10}\left(\lambda_{1}^{2}-\frac{1}{\lambda_{1}}\right)+2k_{1}\lambda_{1}^{2}\left(\lambda_{1}^{2}-1\right)\text{exp}\left(k_{2}{\left(\lambda_{1}^{2}-1\right)}^{2}\right)$$

where the first term represents the elastomer behavior and the second term represents the fiber behavior. The $C_{01}$ term from the Mooney-Rivlin equation is dropped as it mainly affects the behavior of the elastomer at low strains, while the focus of the HGO equation is modeling the strain-stiffening behavior at higher strains. Although the equation is meant to describe aorta, which may be thought of as an embedded fiber-elastomer composite, one of the most important hypotheses of this work is that the equation can still hold for jacketed elastomer tubes where the fabric is not specifically adhered to the elastomer in any way. Thus, Equation (3) was adapted for this scenario such that for the fabric alone, the uniaxial stress equation was

(Equation 4) $$t_{1,fabric}=2k_{1}\lambda_{1}^{2}\left(\lambda_{1}^{2}-1\right)\text{exp}\left(k_{2}{\left(\lambda_{1}^{2}-1\right)}^{2}\right)$$

where $t_{1,fabric}$ is the fabric’s true stress in the direction of elongation (Pa), and $k_{1}$ (Pa) and $k_{2}$ (dimensionless) are the coefficients of interest.

**Figure 3:**
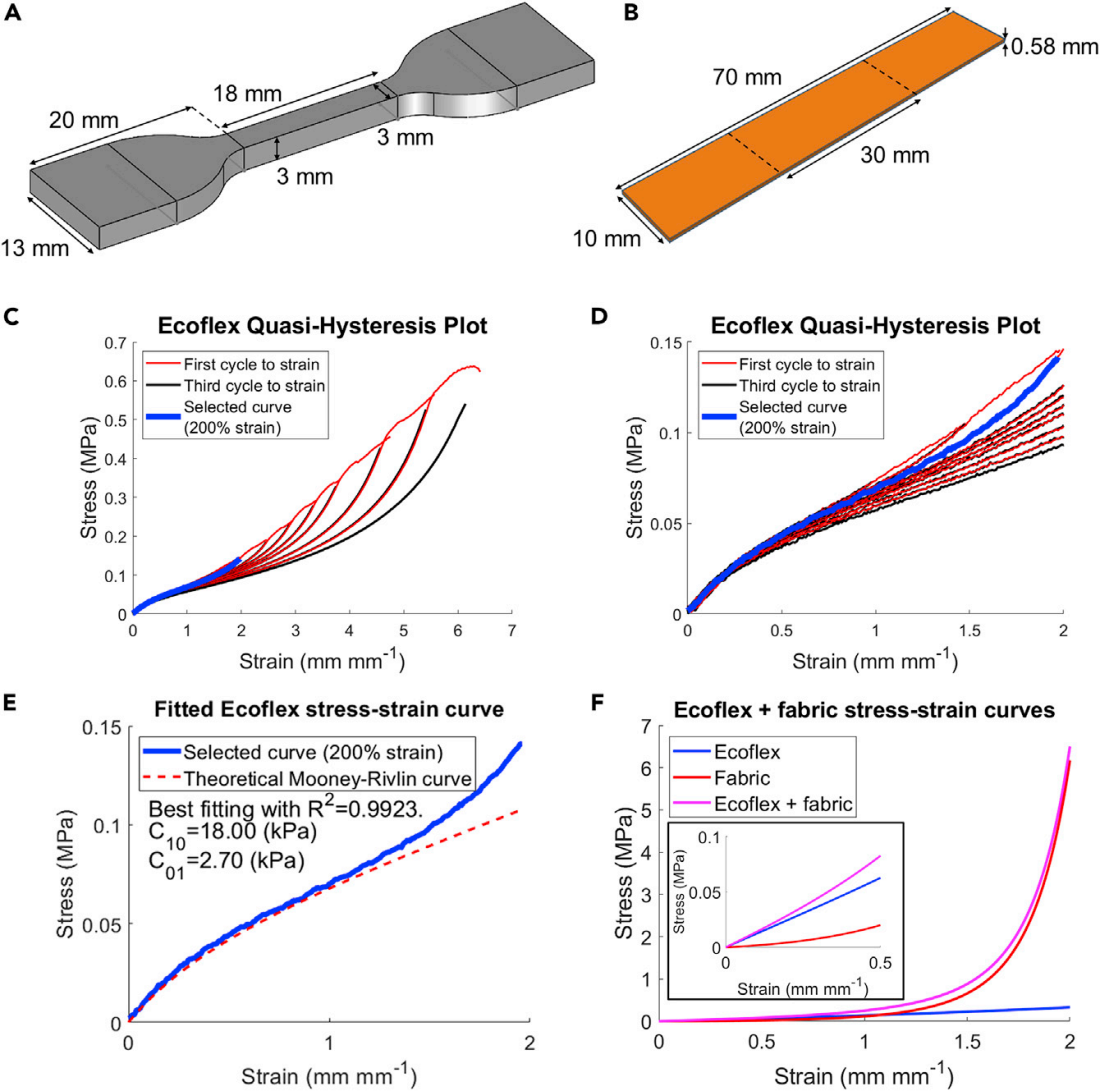
Uniaxial tensile testing (A) Labeled diagram of “dogbone” sample used for tensile testing elastomers. (B) Labeled diagram of strip sample used for testing fabric. (C) Quasi-hysteresis plot of Ecoflex, with selected curve highlighted. (D) Zoomed-in version of (C). (E) Stress-strain plot of selected curve from quasi-hysteresis test, overlaid with theoretical curve produced by coefficients fit to the first 100% strain. (F) Stress-strain curves of Ecoflex, fabric, and Ecoflex-fabric bilayer using fitted coefficients. See also Figures S9–S21.

For uniaxial tensile tests, custom 3D printed molds were used to cast elastomeric “dogbones” for uniaxial tensile testing. The geometry of the dogbone can be seen in Figure 3A. The rayon-spandex fabric was cut into rectangular strips, shown in Figure 3B, and samples with the long edge running parallel to the wale and course directions were both cut in order to evaluate the material anisotropy. However, because it is assumed the tubes will be subject to repeated load-unload cycles when in use, the hysteresis experienced by the material over time must be considered.

Single uniaxial tensile tests to failure were not adequate to determine the material coefficients as they would underestimate the compliance. Here, very subtle change in stiffness due to loading/unloading cycles can cause dramatic effect in the degree of distension, as is illustrated in the supplemental information, Figure S14. Instead, “quasi-hysteresis” tests were conducted, where the samples were subject to multiple load-unload cycles at incrementally larger levels of strain and stress-strain data collected for each. Owing to the limitations of the equipment used, stress-strain data could not be collected when unloading as in a conventional hysteresis test; instead, the quasi-hysteresis plots were formed by overlaying the first and last stress-strain curves when loading to each strain level (Figures 3C and 3D).

Ultimately, one of the curves on each quasi-hysteresis plot was deemed an appropriate approximation of the extent of hysteresis experienced by each material during testing, and the coefficients were selected that would reasonably fit the data to the selected equation. The extent of hysteresis for the elastomers and the fabric was assumed to be equal to that shown in the 200% strain hysteresis curve. This was initially based off a simple approximation; the tubes experienced the most deformation in the circumferential direction, and for all tubes, the maximum pressure tested brought the tube to approximately 200% of its initial diameter. Consequently, the 200% curves eventually were shown to be an excellent benchmark for fitting coefficients that produced accurate simulations, as further explained in the Discussion.

MATLAB’s curve fitting toolbox was then used to apply a least-sum-squares (LSR) regression that could quantify the accuracy of the selected coefficients with an $R^{2}$ value. For elastomers, the coefficients were fit to only the first half of the 200% hysteresis curve (up to 100% strain) as it was deemed their compliance at lower strains was most important to accurately describe (shown in Figure 3E). For the fabric jackets, coefficients were fit to the 200% strain quasi-hysteresis curve of the fabric when tested in the course direction as the circumferential direction of the tube ran parallel with the course direction of the knit; coefficients were fit to the entire strain range of the fabric hysteresis curve in order to accurately describe the strain-stiffening behavior. The material coefficients and associated $R^{2}$ values for the samples undergone the loading/unloading cycles up to 200% strain are tabulated in Table 1. Additional plots of the full quasi-hysteresis curves and theoretical curves formed by the constitutive model coefficients are provided in the supplemental information, Figures S9–S13 and S15–S21.

**Table 1:** Fitted coefficients for all tested materials. All fittings are performed for samples that have undergone loading/unloading cycles up to 200% strain

Material	Material coefficients	R^2^ value
Ecoflex 00-50 (EF)	$C_{10}$ = 18000 Pa, $C_{01}$ = 2700 Pa	*R*^2^ = 0.9923
Dragon Skin 10 SLOW (DS)	$C_{10}$ = 35000 Pa, $C_{01}$ = 3000 Pa	*R*^2^ = 0.9512
Rayon-spandex fabric, course direction	$k_{1}$ = 3336 Pa, $k_{2}$ = 0.0399	*R*^2^ = 0.9987

**Table 2:** R^2^ values of experimental tube distensions and volumes against simulated tube distensions and volumes

Material	R^2^ (Distension)	R^2^ (Volume)
EF	0.9469	0.9332
DS	0.9856	0.9749
EFF	0.9862	0.9857
DSF	0.9702	0.9617

A fabric-jacketed tube can therefore be assumed to have a uniaxial tensile stress equation equivalent to Equation (3), using a combination of the coefficients in Table 1. The associated stress-strain curve is shown in Figure 3F. As expected, the elastomer material response is dominant at the lower strains while the fabric quickly becomes responsible for most of the stiffness at higher strains.

### Finite element simulation results

**Figure 4:**
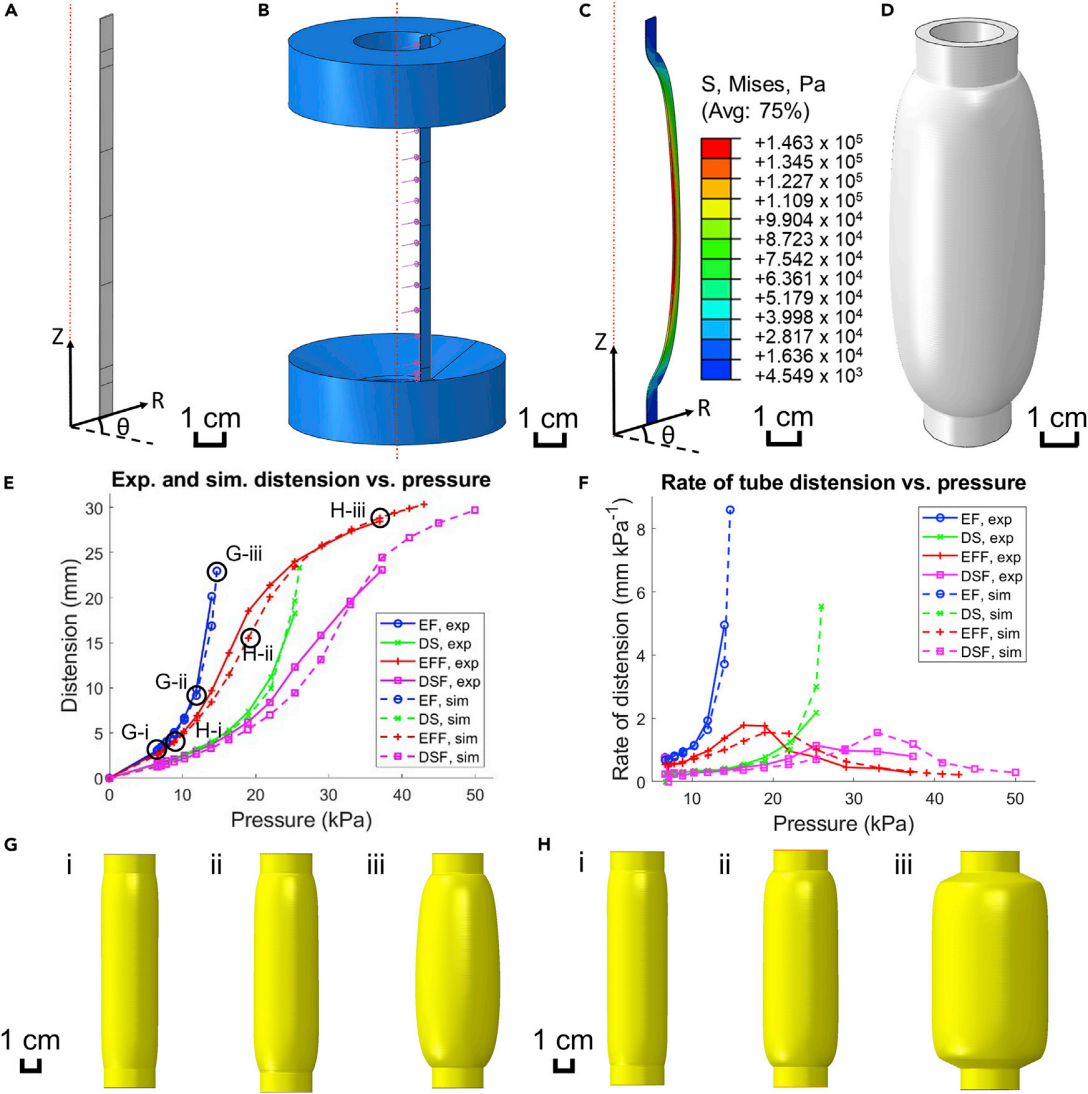
Finite element analysis of elastomeric tubes experiencing hydraulic internal pressure (A) ABAQUS-modeled “slice” of cylindrical tube wall, with coordinate system labeled. Scale bar: 1 cm. (B) Assembly of the tube slice and analytical rigid rollers. Scale bar: 1 cm. (C) Distended tube slice with color mapping for von Mises stress. Scale bar: 1 cm. (D) Full tubular representation of the distended slice in (C). Scale bar: 1 cm. (E) Plot of simulated radial tube distension (mm) vs. pressure (kPa), overlaid with experimental data; EF = Ecoflex, DS = Dragon Skin, EFF = Ecoflex + fabric, DSF = Dragon Skin + fabric. (F) Plot of simulated rate of distension (mm kPa^−1^) vs. pressure (kPa), overlaid with experimental data. (G) Finite element models of bare Ecoflex tubes at pressures of (i) 6.52 kPa, (ii) 10.25 kPa, and (iii) 13.99 kPa. Scale bar: 1 cm. (H) Finite element models of jacketed Ecoflex tubes at pressures of (i) 8.84 kPa, (ii) 18.99 kPa, and (iii) 36.95 kPa. Scale bar: 1 cm. See also Figures S23–S38.

Three-dimensional finite element models were then created in ABAQUS to replicate the hydrostatic testing for all tested tubes. Because the base design of the tubes was axisymmetric, only a thin “slice” of each tube was modeled to save computational power (Figure 4A). Different models were created to represent EF, DS, EFF, and DSF tubes, each using the relevant coefficients from Table 1 to define the material. Hydrostatic loads were applied to the inner wall of the tubes with analytical rigid rollers used to represent the clamps in the physical experiment (Figure 4B). The load was gradually increased to reflect each pressure level to which the physical tubes were subject during the experiment, plus one or more extra levels beyond the experimental maximum to monitor the continued evolution of the distension trends.

After each step in the simulation, the tube slice took on a distended shape which could be color-mapped to show the distribution of stress, strain, or other material properties (Figures 4C and S22). Performing a 360° sweep of this shape allowed for a better visualization of the distended profile of the entire tube (Figure 4D). Analysis of the deformed models allowed for the calculation of maximum distension at each simulated pressure level and rate of distension per unit pressure increase; the same equations in the hydrostatic pressure testing results section were used, where maximum distension was defined as the average difference between final and initial diameter over the middle 3 cm of the tube. These results are plotted in Figures 4E and 4F, with profiles of distended EF tubes at various pressures shown in Figure 4G:i-iii and distended EFF tubes at various pressures in Figure 4H:i-iii. Additional data from the simulations are provided in the supplemental information, Figures S23 and S24.

Clear trends emerge from the ABAQUS simulation results. The unjacketed tubes show exponential increases in distension and rate of distension as pressure increases, while the jacketed tubes have a sigmoid-shaped distension curve due to the strain-stiffening behavior of the fibers. Additionally, increasing the simulated pressure range allows for the full realization of the self-regulation behavior observed in the jacketed tubes; as seen in Figure 4F, the rate of distension continues decreasing to nearly zero as hydrostatic pressure increases further.

## Discussion

### Comparison of experimental and simulated results

Figures 4E and 4F show that there is good agreement between physical experiment and numerical simulation for the distension trends of all tested tubes. The distension-pressure curves for the unjacketed elastomer tubes experience are exponential (continually increasing distension rates) whereas for the jacketed tubes they are sigmoidal (distension rates that peak and then fall). The results may be compared quantitatively via the use of parity plots, whose results are summarized in Table 2. All comparison plots are available in the supplemental information, Figures S27–S30 and all parity plots are available in the supplemental information, Figures S31–S38.

**Figure 5:**
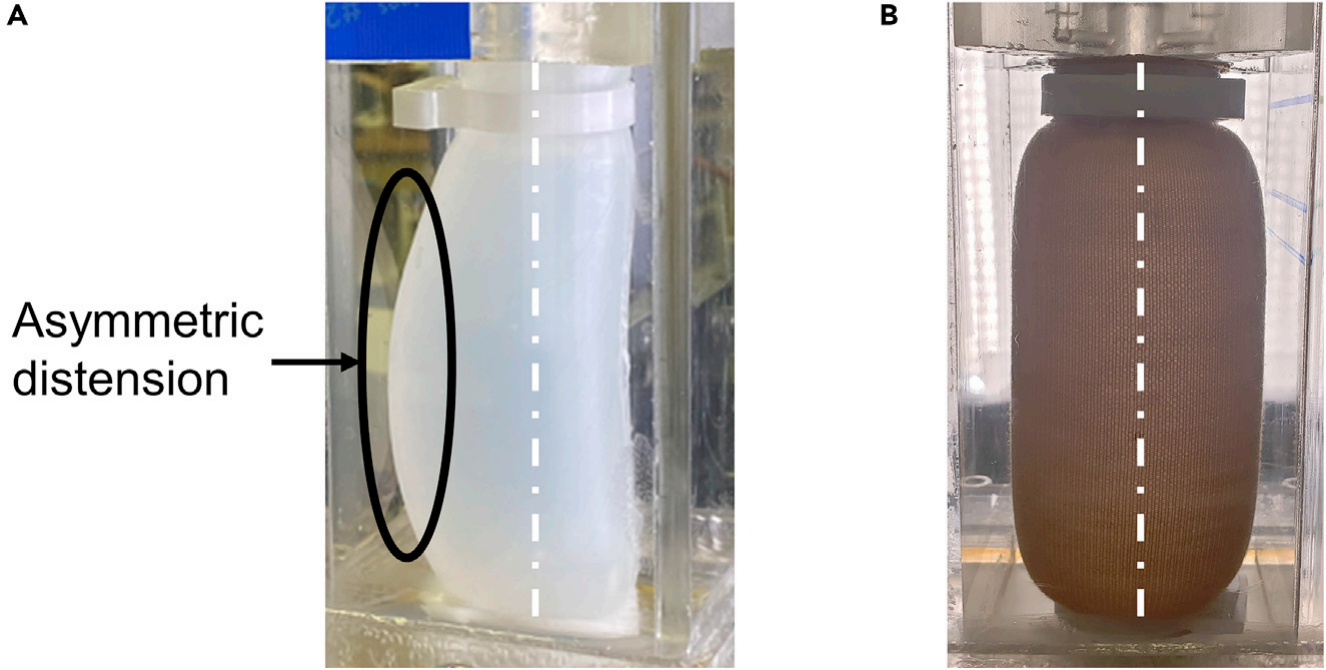
“Aneurysm” in elastomeric tubes and its suppression by applying strain-stiffening jacket (A) Bare Ecoflex tube at high pressure displaying marked asymmetric distension. (B) Jacketed Ecoflex tube at high pressure. Approximated lines of symmetry are overlaid on both images. See also Figures S5–S8.

Predictably, the asymmetric distension observed in the elastomeric tubes at high pressures did not occur in the simulations. This discrepancy may be reconciled by considering the real-life limitations of the tubular cast geometry. Inevitably, any cast elastomeric tube will contain localized inhomogeneities in physical properties (wall thickness), material properties (elastic modulus), or stress distribution (residual stresses leftover from the fabrication process). In any of these cases, there will be small areas of elevated stress on the tube wall which would result in localized regions of greater distension. Repeated over many pressure tests, this could accelerate the hysteresis behavior in these localized regions, creating regions of significantly lower compliance. This would catalyze a cascading effect that may be responsible for the “aneurysm” shown in Figure 5A.

In fact, a similar phenomenon has been observed and published in literature pertaining to internal pressurization of incompressible hyperelastic tubes: given enough pressure, eventually a mostly uniform deformation will give way to a strongly localized bulging somewhere along the length of the tube with deloading everywhere else (Kyriakides and Chang, 1991; Pamplona et al., 2006; Ye et al., 2020). Much attention has been given to this phenomenon including both physical experiments and FEA simulations, and it has been observed that the degree of inhomogeneity required to initiate this deformation mode can be extremely slight (Ye et al., 2020).

Conversely, if such defects are present in a jacketed elastomeric tube, the strong strain-stiffening behavior of the fabric may act as an inhibitor of the uneven distension that would occur in bare elastomeric tubes. Indeed, it is known that one of the important physiological roles of collagen fibers in aorta is to provide structural support and suppress aneurysm (Roveri et al., 1978; Belz, 1995; Holzapfel et al., 2000). Figure 5B shows a clear difference in distension profiles between an unjacketed and jacketed elastomeric tube near the upper limits of their tested pressures; image analysis is able to quantify this asymmetry which is further explained in the supplemental information, Figures S5–S8. The observed self-regulation behavior in jacketed elastomeric tubes may play a pivotal role in several of its applications.

### Outlook

Ultimately, this work is a proof of concept for a material phenomenon using a limited set of experiments. By carefully altering the design of the tubing to induce onset of self-regulation at a particular pressure or distension, it may be used for a myriad of purposes discussed in the Introduction. In the future, the limits of the tunability of the material response should be further investigated.

Within the contexts of this experiment, the simplest change that could be made is changing the circumference of the fabric jacket. Increasing the circumference (introducing slack into the jacket) would delay the onset of the self-regulation response, whereas decreasing the circumference would introduce pre-stretch in the fabric (essentially changing its material coefficients) and hasten the onset of self-regulation. Similarly, by printing a new mold design, the base elastomeric tubing could be customized by changing its radius, length, or wall thickness.

To produce tailored material responses with greater complexity, the use of metamaterial designs may be an option. Kirigami, the Japanese art of cutting and folding paper to produce repeated patterns (Ma et al., 2017), could easily be induced in the fabric jacket to control the distension profile of the jacketed tube. Strategically cut regions in the fabric jacket would create localized regions of increased compliance in the tube, which can then be controlled carefully in accordance with the working conditions to produce highly specific material responses. A similar concept was explored in a work by Belding et al. (2018) which used polypropylene sheeting as an outer jacket instead of knitted fabric.

**Figure 6:**
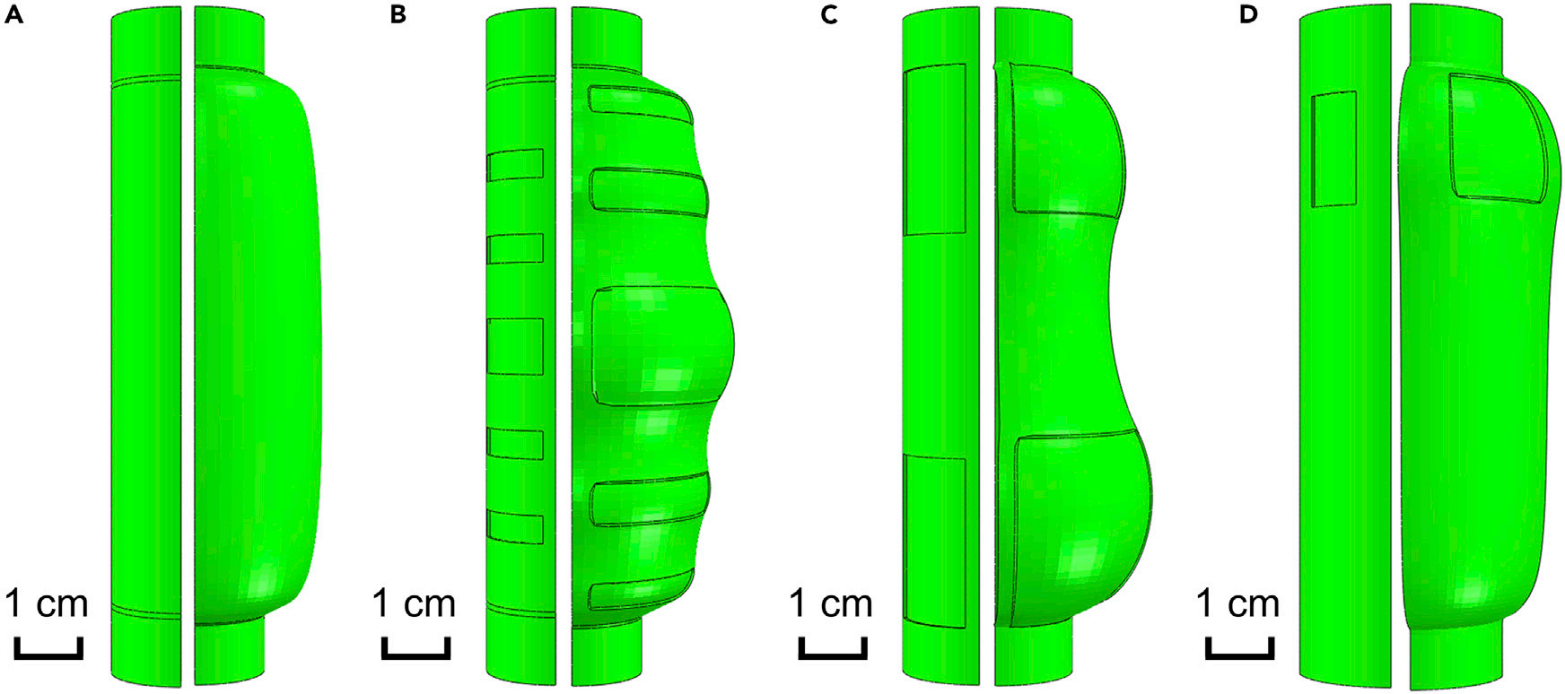
ABAQUS models of fabric-jacketed elastomeric tubes with kirigami patterns cut into the fabric (A) Control group; jacketed tube, undeformed (left) and subject to 25 kPa hydrostatic pressure (right). (B) Jacketed tube with kirigami pattern to make middle of tube distend preferentially, undeformed (left) and subject to 25 kPa hydrostatic pressure (right). (C) Jacketed tube with kirigami pattern to make top and bottom of tube distend preferentially, undeformed (left) and subject to 18 kPa hydrostatic pressure (right). (D) Jacketed tube with kirigami pattern to make top of tube distend preferentially, undeformed (left) and subject to 20 kPa hydrostatic pressure (right). Scale bar: 1 cm

Additional ABAQUS simulations are designed to illustrate this concept. Figure 6A depicts a jacketed Ecoflex tube in its undeformed state and deformed state under 25 kPa hydrostatic pressure. Figures 6B–6D illustrate the same concept, but with circumferential kirigami patterns cut into the fabric. In ABAQUS, the portions of the tube below the cuts are defined as bare Ecoflex while the rest is defined to be the Ecoflex-fabric composite. In Figure 6B, the cuts are biased toward the center to encourage greater distension near the middle of the tube; in Figure 6C, the cuts are biased toward the top and bottom to encourage less distension near the middle of the tube; and in Figure 6D, the single are biased toward the top to encourage a more balanced distension along the entire length of the tube. The resulting distension profiles show that these goals are well achieved. The altered distension profiles could prove beneficial in some applications, e.g. if the distended shape allows for quicker fluid flow during depressurization.

### Conclusion

There are three main conclusions to this work. First, we present a design for jacketed elastomeric tubes that display self-regulation behavior at elevated hydrostatic pressures. Specimens were manufactured using 93%–7% rayon-spandex blend knit fabric and cast elastomeric tubes made of both Ecoflex 00-50 and Dragon Skin 10 SLOW. The jackets were created by sewing rectangular pieces of fabric along their long edges to form closed loops, then sliding them over the elastomeric tubes. The distension-pressure curves of bare elastomeric tubes were exponential; the tubes experienced rapid deformation with increasing pressure which quickly led to asymmetric distension. Conversely, jacketed tubes had sigmoidal distension-pressure curves and largely symmetric distension profiles; the expansion of the knit fibers led to strain-stiffening, which is the basis of the self-regulation response.

Second, in addition to a series of hydrostatic pressure tests, finite element models were successfully developed in ABAQUS to validate, and expand on, the trends seen in the physical experiments. Material behavior in the simulation was defined using hyperelastic constitutive models, which required numerical coefficients; these coefficients were obtained by fitting them to uniaxial tensile test data of the materials of interest. In order to accurately represent the increase in compliance undergone by the elastomer after many load-unload cycles, a single uniaxial tensile test of a pristine sample to failure could not be used. Instead, a quasi-hysteresis test was conducted (loading cycles measured only), and the coefficients were fit to a single curve on the overall plot that was deemed to best represent the extent of hysteresis experienced by the material. Simulations were successful in replicating the experimental results, both in terms of overall trends and numerical values.

Finally, the base design of the tube is highly tunable and customizable, making it potentially suitable for a wide variety of applications. Prospective applications for the presented design span the biomedical, soft robotic, and industrial sectors. Small changes to the design such as altering tube wall thickness, fabric jacket circumference, or knit fabric orientation may be made to yield simple, uniform changes to the material behavior. More complex alterations, such as cutting kirigami designs in the jacket, may be used to induce more complex material responses.

### Limitations of study

Limitations of the pressure testing rig bounded the maximum pressure that the elastomeric tubes could be tested at. For example, the jacketed Ecoflex tube could not be tested beyond a pressure of 36.95 kPa due to the walls of the deformed tube threatening to hit the walls of the compliant chamber, and the jacketed Dragon Skin tube could not be tested beyond a pressure of 37.30 kPa due to a leak occurring elsewhere in the setup.

For each of the tested tube compositions, n = 1 due to the difficulty of properly fabricating the tubes and running the hydrostatic pressure tests on the flow loop.

## Data statement

The research data required to reproduce the results in this manuscript are available upon request. Contact Prof. Hyun-Joong Chung at chnug3@ualberta.ca for more information.

## STAR★Methods

### Key resources table

**Table:** 

REAGENT or RESOURCE	SOURCE	IDENTIFIER
**Chemicals, peptides, and recombinant proteins**		

Ecoflex 00-50 resin	Smooth-On, Inc	N/A
Dragon Skin 10 SLOW resin	Smooth-On, Inc	N/A

**Deposited data**		

Raw and analyzed data	Lead author	chung3@ualberta.ca

**Software and algorithms**		

ABAQUS 2020	Dassault Systèmes	https://www.3ds.com/products-services/simulia/products/abaqus/
MATLAB R2021a	MathWorks	https://www.mathworks.com/products/matlab.html

**Other**		

93-7% rayon-spandex blend knit fabric	Télio	N/A

### Resource availability

#### Lead contact

Further information and requests for resources and data discussed in this work should be directed to and will be fulfilled by the lead contact, Dr. Hyun-Joong Chung (chung3@ualberta.ca).

#### Materials availability

The components of the materials studied in this work are publicly available for purchase. Ecoflex™ 00-50 and Dragon Skin™ 10 SLOW resins can be purchased from Smooth-On, Inc. (U.S.), while 93-7% rayon-spandex blend knit fabric can be purchased from Télio Fabrics (Canada).

### Method details

#### Materials of interest

Ecoflex 00-50 and Dragon Skin 10 SLOW are platinum-catalyzed RTV silicone elastomers obtained from Smooth-On Inc. (US). In liquid resin form, EF and DS have 2 components that were mixed in a 1:1 ratio and degassed before being poured into molds to cure in their desired shape. Knitted fabric (93% rayon and 7% spandex) was sourced from Télio (Canada). These materials were both previously identified in work by Zhalmuratova et al. (Zhalmuratova et al., 2019) for their ability to replicate the properties of human aorta when used in composites together, and RTV silicones in particular were noted for their ease of use in a wet *in vivo* environment and previous uses within biomedical applications. Additional material properties of the elastomers are provided in Table S2.

#### Hydrostatic pressure testing of elastomeric tubes

##### Sample Preparation

Elastomeric tubes were manufactured by pouring mixed and degassed liquid resin into a 3D printed cylindrical mold and being allowed to cure for a minimum of 3 h. The mold produced tubes with an OD of 27.05 mm and had a centre insert to produce an inner diameter of 19.05 mm.

For tests involving jacketed tubes, the fabric was cut into rectangular pieces and sewn to form ‘jackets’ 12 cm in length and 8 cm in circumference, with the stiffer wale direction parallel to the longitudinal axis of the tube and the more compliant course direction running along the circumference. This orientation was originally proposed in previous work by Zhalmuratova et al. (Zhalmuratova et al., 2019) where it was determined the wale direction should run parallel to the longitudinal axis of the tube to better replicate the properties of the collagen fibers in the aortic adventitia. The fabric jackets were then pulled over the elastomeric tubes, which increased the initial OD to 28.21 mm as the fabric was 0.58 mm thick on average. A schematic of the compliant tube is shown in Figure 1B.

##### Pressure testing setup

A custom-built pressure-testing rig was used to test the tubes under hydrostatic pressure. A schematic of the tester is shown in Figure 2A. The centrifugal pump used is a Jostra AB RFC 20-970 and the flowing fluid is water at ambient temperature. The compliance chamber housed a length of elastomeric tubing with the ends clamped in place as shown in Figure 2B, and fluid flowed into it via the centrifugal pump. the check valve beneath the tube was closed to prevent fluid flow past the compliant chamber, achieving hydrostatic conditions.

Before testing began, a degree of pre-stretch was applied by running the centrifugal pump at increasingly higher speeds until severe, asymmetric distension of the tube occurred (jacketed tubes were pre-stretched without the jacket on). The pressure was then relieved and the tube allowed to return to its undeformed state. For the actual tests, centrifugal pump speed was increased in 200 rpm increments and the corresponding pressure was measured by a tap just below the compliant chamber. As fluid flowed into the tube, it distended and a camera facing the compliance chamber took pictures of the distended tube’s profile. The camera was a Basler AG acA800-510um, which was leveled prior to testing along with the compliant chamber to ensure one end of tube was not tilted forward in the images.

##### Image analysis

Prior to taking the experimental images, a ‘calibration’ elastomeric tube cast with the same mold as the experimental tubes was hooked up to the compliant chamber and imaged in its undeformed state. This tube had markings drawn on its surface which were 1 cm apart and made to face the camera head-on. Since all of the images taken by the camera were the same size (800 × 600 pixels), this could be used as a calibration to correspond pixels to centimeters for later analysis.

Figure 2C is an example of an image taken by the camera facing the compliant chamber. The images of the distended tubes were assessed to measure the amount of radial deformation experienced by the tube and the volume increase at each pressure level. A MATLAB program was written that binarized these grayscale images and calculated the ‘width’ of the tube along its entire length.

The MATLAB program calculated the maximum experimental distension $\delta_{exp}$ of the tubes by averaging the distended tube’s outer diameter $D_{f}$ over the middle 3 cm of the image and subtracting the undistended outer diameter $D_{i}$. More specifically, the program split the image into ‘rows’ of pixels and measures the width of the distended tube, in pixels, for each row. The measurements were later converted to SI units using the calibration. This is described in Equation (5):

(Equation 5) $$\delta_{exp}=\frac{h_{pixel}}{j_{max}}\sum_{j=1}^{j_{max}}\left(D_{f,\;j}-D_{i}\right)$$

where the range [1, $j_{max}$] represents the number of measurements taken in the middle 3 cm of the image (corresponding to the number of pixels in 3 cm of the image), $D_{f,j}$ is the final outer diameter measurement at point *j* (px), $D_{i}$ is the initial outer diameter of the tube (px), and $h_{pixel}$ is the calibration i.e. the height of one pixel (mm px^−1^). $D_{i}$ has a constant value of 27.05 mm for unjacketed tubes and 28.21 mm for jacketed tubes.

Similarly, the volume of the distended tubes was calculated by taking row-by-row measurements of the tube’s width for the entire image except for the middle 3 cm. However, the images only measured the middle 8 cm of the 12 cm tubes. Since 1 cm on either end of the tube is clamped (and is ignored in the volume calculation), there was still an additional 1 cm of the distended tube wall on either end of the image that was not shown. To account for this, an extrapolation method was used in which the tube diameter was assumed to return to the undistended outer diameter $D_{i}$ linearly over the missing 1 cm. The formula to calculate the volume is then given in Equation (6).

(Equation 6) $$V_{exp}=\sum_{k=1}^{k_{max}}\frac{\pi }{4000}\;D_{f,k}^{2}h_{pixel}-V_{walls}$$

$D_{f,k}$ is the distended outer diameter of the tube for row *k* in the image (mm), $V_{exp}$ is the experimental volume (mL), $k_{max}$ is the height of the image plus the extrapolated regions in pixels (px). $V_{walls}$ is the volume of the tube walls (mL), which are assumed to be incompressible and can have their volume calcluated using Equation (7).

(Equation 7) $$V_{walls}=\frac{\pi h}{4000}\;{\left(D_{i}-d_{i}\right)}^{2}$$

where $D_{i}$ is the undistended outer diameter, $d_{i}$ is the undistended inner diameter, and *h* is the working height of the undeformed tube (all mm). For all tested tubes, $d_{i}$ = 19.05 mm and *h* = 100 mm.

Unit rate of distension $\dot{\delta }$ (mm kPa^−1^) and unit rate of expansion $\dot{V}$ (mL kPa^−1^) can also be calculated using Equations (8) and (9). It must first be noted that for each tested tube there are *N* distension and volume measurements taken, each corresponding to one of *N* level of pressure *P* (kPa).

(Equation 8) $${\dot{\delta }}_{n}=\frac{\delta_{n}-\delta_{n-1}}{P_{n}-P_{n-1}}$$

(Equation 9) $${\dot{V}}_{n}=\frac{V_{n}-V_{n-1}}{P_{n}-P_{n-1}}$$

where *n* is the point of interest and is in the range [2, *N*]. Plots of the experimental data for tube distension, rate of distension, tube volume, and rate of volumetric expansion are shown below. Ultimately, the volume plots follow the same trends seen in the distension plots; unjacketed tubes undergo exponential volume increase and steadily increasing rate of volumetric expansion with increasing pressure, whereas jacketed tubes experience a plateau in volume increase due to the self-regulation behavior.

#### Uniaxial tensile testing of materials

Stress-strain behavior of elastomer and fabric samples was measured using an Instron 5943 uniaxial tester. The main purpose of these tests was to obtain constitutive modelling coefficients for each material that could be used in the subsequent finite element simulations. This required knowledge of the material hysteresis behavior, such that the increase in compliance with load history could be reflected in the selected coefficients; a single uniaxial tensile test to failure of a pristine sample of each material would be insufficient as it would severely overestimate the stiffness of a sample that had been aged from previous loading-unloading cycles. For example, an aorta-substitute tube in an EVHP device undergoes cyclic deformations during operation; this results in the deviation of the tube material’s stress-strain behavior from that measured from the virgin material.

Conventional cyclic hysteresis testing, in which the tester automatically performs a given number of load-unload cycles at a precisely controlled rate and continuously measures the output, was not possible with the instrument used (unloading rate is automated). Instead, a ‘quasi-hysteresis’ test was designed where the sample was stretched to a given maximum strain 3 times before the maximum strain increased by 50% (100% once max strain reached 400%), but the apparatus recorded the stress/strain data for each test during loading only. For every 50% of strain experienced by the sample in the previous loading cycle, 30 s of relaxation time was allowed between loading measurements.

The width, thickness, and gauge length of the elastomeric samples were measured using a caliper. The loading was performed at an extension rate of 0.1 mm mm^−1^ s^−1^ based on previous literature that conducted similar tests (Creton, 2017). The stress-strain plots for the load ‘cycles’ could then be superimposed to see the increase in compliance as the quasi-hysteresis test progressed, as shown in Figure 3C and closer up in Figure 3D.

#### Finite element modeling of elastomeric tube distension

After obtaining the material coefficients, finite element modeling was performed in ABAQUS (Dassault Systèmes SE). To minimize computation time, each tube was modeled as a longitudinal ‘slice’ encompassing a portion of the circumference equivalent to the thickness of a single element (approximately 0.5 mm). The tube slices were defined in a cylindrical coordinate system as shown in Figure 4A. The entire part was meshed using a seed of 0.5 mm with 8-node linear brick, hybrid (C3D8H) elements. The equation solver operated using the Direct method and the Full Newton solution technique. Each step had a time period of 1, an initial increment size of 0.01, a minimum increment size of 0.000001 and a maximum of 10000 increments.

The tube slice’s material behavior was defined to be hyperelastic and governed by the coefficients obtained from the uniaxial tensile tests. For the jacketed tubes, the material was assumed to be a single-layer, homogeneous fiber-elastomer composite with fiber families running in the circumferential and longitudinal directions. Since most of the distension in the fabric jacket was circumferential, it is assumed that most of the material response is governed by the stiffness of the fabric in the course direction; therefore, the uniaxial coefficients obtained from the fabric’s course direction tensile test were assumed to be representative of the fabric’s behavior in the simulation.

For boundary conditions, the ‘cut faces’ of the tube slices were restricted from moving in the circumferential direction; this essentially functioned as a cyclic symmetry condition. The top and bottom faces of the tube slice were restricted from moving in the longitudinal direction to represent the fixed ends due to clamping. Most notably, analytical rigid parts at the top and bottom of the tube constrained it from expanding radially, which were loosely representative of the real-life clamps used to hold the tube in place in Figure 2B. Contact between the tube and the clamps was defined to be frictionless and the clamp’s cross-sectional geometry was designed to maximize the stability of the simulation without greatly affecting the results. The finite element assembly is shown in Figure 4B.

The pressure load was defined as hydrostatic, applied across the entire inner face of the tube, and its magnitude was increased with each sequential step of the analysis to reflect each of the experimental pressures. Since each hydrostatic pressure in the physical experiments was based off a measurement taken just below the bottom of the tube, for the simulations these pressures were assumed to apply to the very bottom of the tube (z=0) and decrease with increasing *z* due to gravity effects in accordance with Equation 10.

(Equation 10) ΔP=ρ⋅g⋅Δz

where ρ=999 g m^−3^, g=9.81 m s^−2^, and Δz = 0.12 m (the length of all tested compliant tubes). Equation (10) yields that the gravity effects were responsible for approximately 1.2 kPa of head loss up the length of the tube.

The distended finite element tube slice is pictured in Figure 4C, the face of which could be swept to form a distended tube (Figure 4D). After running the simulation, measurements of maximum tube distension and distended tube volume were calculated for each tested pressure.

Maximum tube distension $\delta_{sim}$ (mm) was calculated for the simulations using a similar process to how the experimental distension values were found. First, history outputs were specified in ABAQUS to measure the radial displacement and z-coordinates (position on the longitudinal axis) for all nodes along one outer edge of the tube slice. Next, a MATLAB program was written that averaged the radial displacement of the middle 3 cm of the tube, from which the maximum distension could be easily calculated by Equation (11).

(Equation 11) $$\delta_{sim}=\frac{1}{j_{max}}\sum_{j=1}^{j_{max}}\left(D_{f,\;j}-D_{i}\right)$$

where $j_{max}$ is the number of nodes contained within the middle 3 cm of the simulated tube, $D_{f,j}$ is the outer diameter at node *j* and $D_{i}$ is the theoretical outer diameter of the tube. Additionally, tube volume was calculated using an integration approximation method. The volume approximation was made by summing many thin volumes of revolution with trapezoidal cross-sections as shown in Equation (12):

(Equation 12) $$V_{sim}=\sum_{k=1}^{k_{max}-1}\left(\frac{\pi }{12000}\left(D_{f,k+1}^{2}+D_{f,k+1}D_{f,k}+D_{f,k}^{2}\right)\left(z_{k+1}-z_{k}\right)\right)-V_{walls}$$

where $V_{sim}$ is the volume (mL), $D_{f,k}$ is the outer diameter at node *k* (in mm, noting there are $k_{max}$ nodes in the region of interest), and $z_{k+1}$, $z_{k}$ are the z-coordinates of nodes k+1 and *k* (mm). $V_{walls}$ retains the same definition as in Equation (7). Finally, unit rate of distension and unit rate of expansion per unit increase in pressure could be calculated for the simulations using the same method as the experimental results (Equations 8 and 9).

### Quantification and statistical analysis

There is excellent agreement in the numerical values of both distension and volume over the entire tested pressure range. This is quantifiable by using a least-squares reduction to determine R^2^ values, as listed in Table 2. All parity plots are available in the supplemental information, Figures S31–S38.

## Data Availability

The data and the material that support the findings of this study are available from the lead contact, Dr. Hyun-Joong Chung (chung3@ualberta.ca) upon reasonable request.

## Supporting citations

The following reference appears in the Supplemental Information: Bergstrom et al., 2015; Rivlin, 1948

## Supplemental information

Document S1. Figures S1–S38

Figure360. An Author Presentation of Figure 2
