# Supplementary material for: Elastomeric tubes with self-regulated distension
Source: iScience. 2022 May 6;25(6):104369. doi: 10.1016/j.isci.2022.104369 (PMC9126797; doi:10.1016/j.isci.2022.104369)
Supplement: Document S1. Figures S1–S38 [file mmc1.pdf]

iScience, Volume *25*

Supplemental information

Elastomeric tubes with self-regulated distension

Nathan Jen, Guilherme M. Bessa, Kaelyn Nicolson, Jianliang Xiao, David S. Nobes, and Hyun-Joong Chung

Supplemental Information

SI - Hydrostatic Pressure Testing

In Figure 2, plots of tube distension and rate of tube distension vs. hydrostatic pressure were included. They are featured below, along with plots of tube volume and rate of volumetric expansion vs. hydrostatic pressure, in Figures S1-S4.

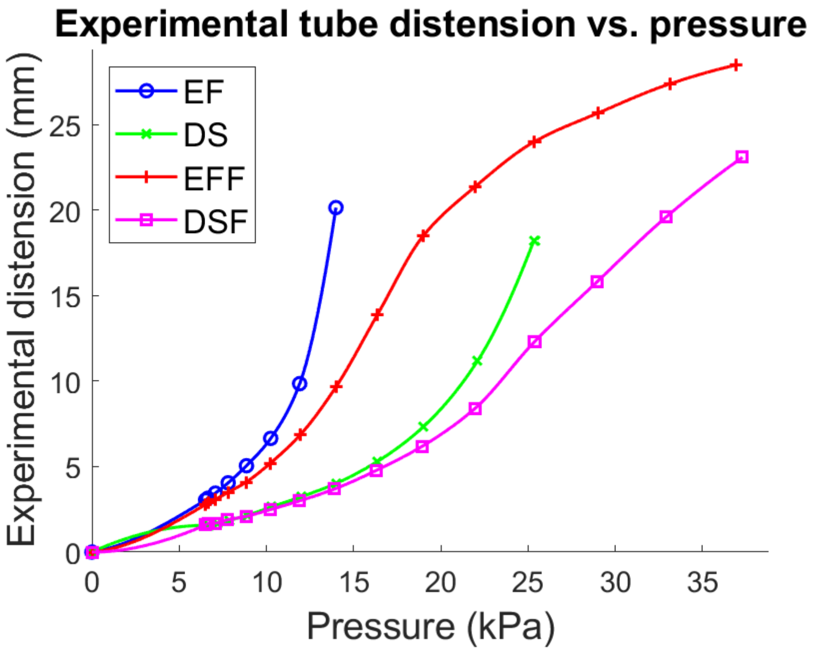

Figure S1: Experimental distension vs. pressure plot, related to Figure 2.

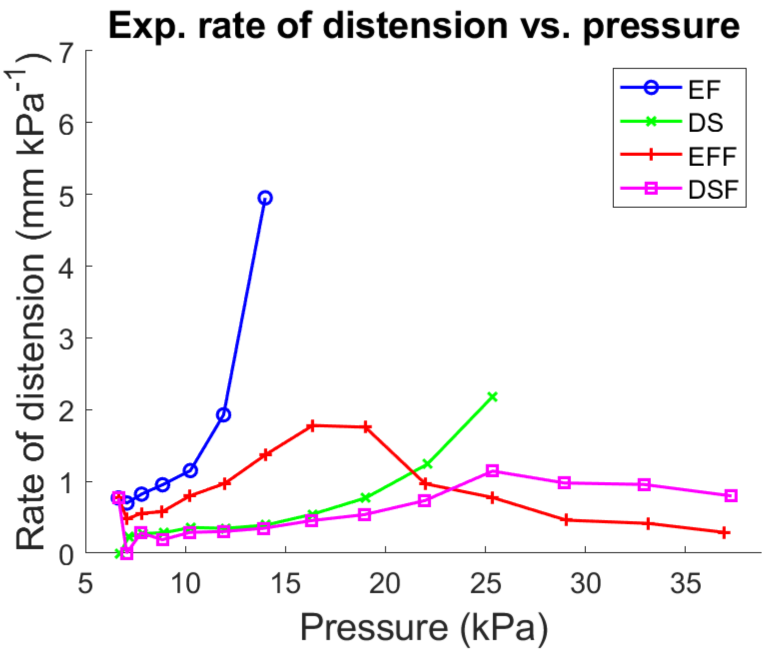

Figure S2: Experimental rate of distension vs. pressure plot, related to Figure 2.

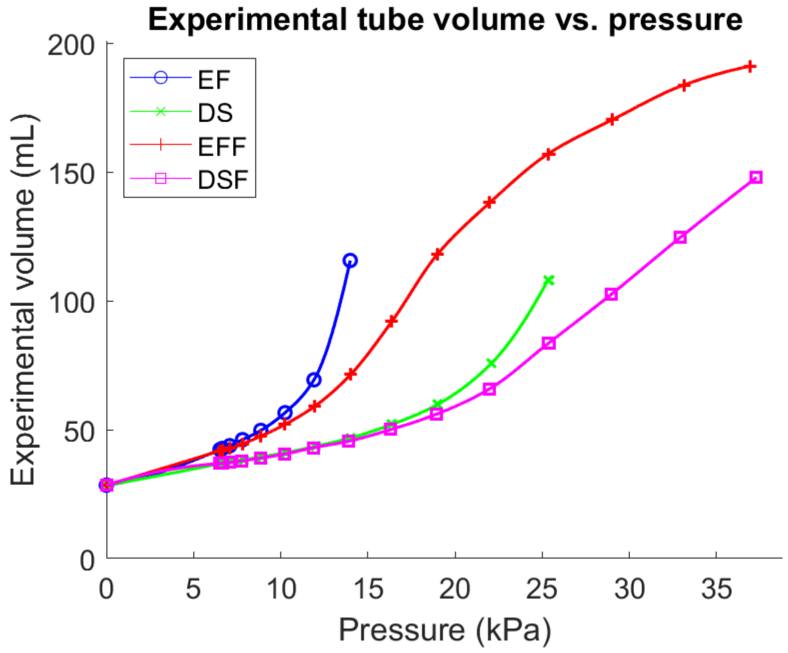

Figure S3: Experimental volume vs. pressure plot, related to Figure 2.

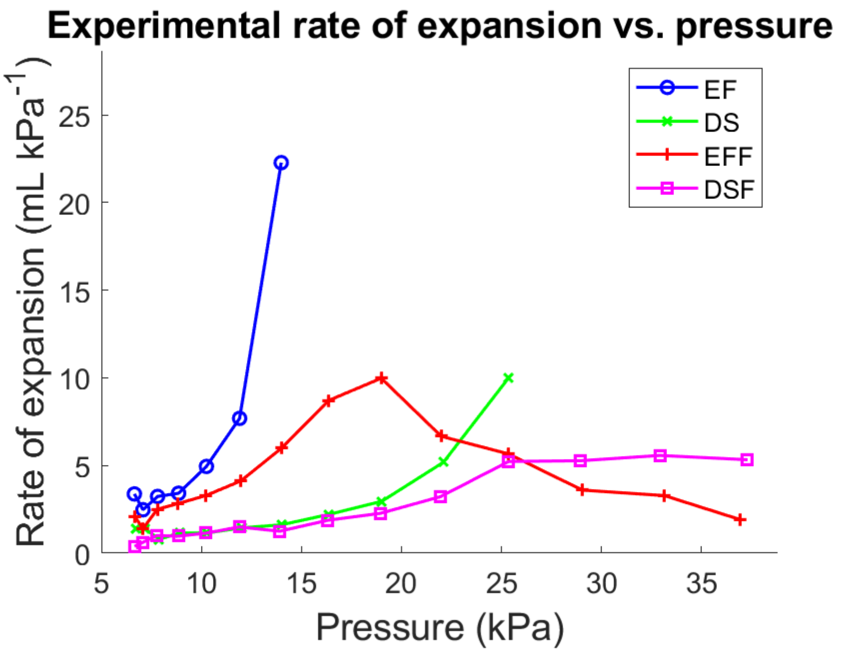

Figure S4: Experimental rate of expansion vs. pressure plot, related to Figure 2.

In the Discussion and in Figure 5, the increased symmetry of the distended tube profile that occurred with the addition of the fabric jacket was mentioned qualitatively. However, a simple quantitative measure can also be applied by use of the mean symmetry index (SI), which is defined in Equation S1.

$$SI = \frac{1}{m_{max}} \sum_{m=1}^{m_{max}} \frac{R_{f,m,right}}{R_{f,m,left}} \tag{S1}$$

where $R_{f,m,right}$ is the right outer radius value of row m of pixels for an image of a distended tube (that is, the outer radius value traveling to the right of the vertical axis of symmetry of the undistended tube), $R_{f,m,left}$ is the left outer radius value, and m_{max} is the number of rows evaluated. Obviously, the image of a distended tube is more symmetric the closer the value of SI is to unity. Therefore, Figures S5-S8 can be used to quantitatively evaluate the symmetry of elastomeric tubes at high pressures. Note that the extrapolated and interpolated radius measurements of the tubes were not used for the purposes of the SI calculations (so $m_{max} =$ 750); however, their exclusion is not expected to have a significant impact.

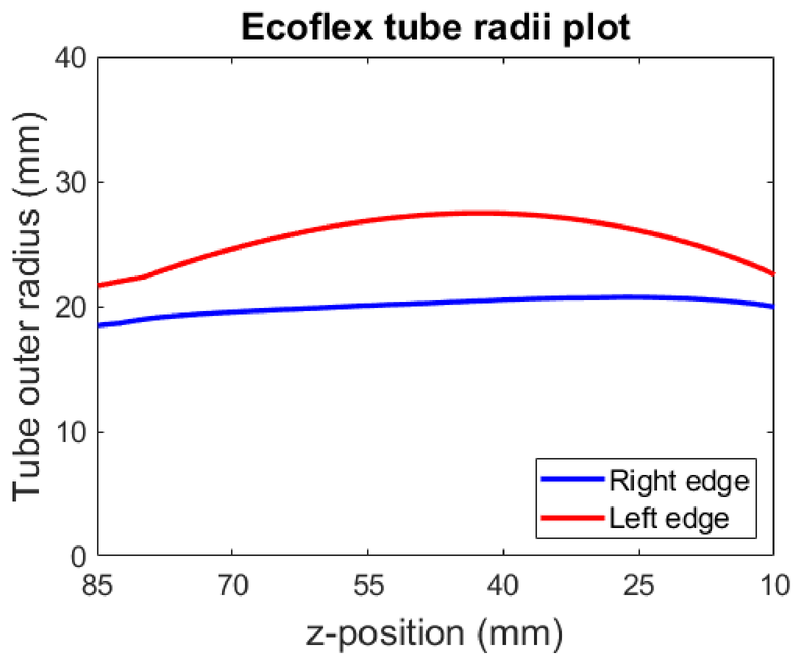

Figure S5: Radius measurement along the edges of a bare Ecoflex tube pressurized at 13.99 kPa, related to Figure 5.

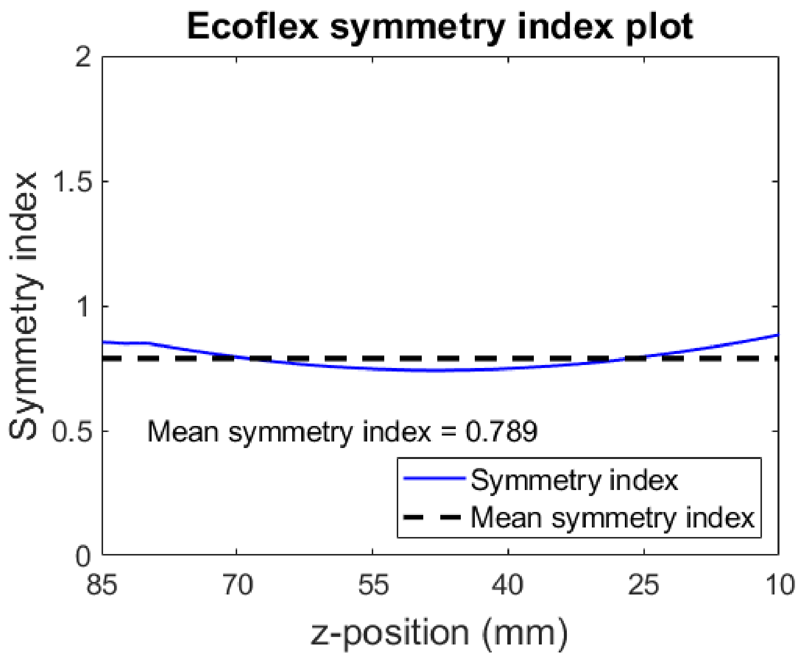

Figure S6: Symmetry index measurement of bare Ecoflex tube pressurized at 13.99 kPa, related to Figure 5.

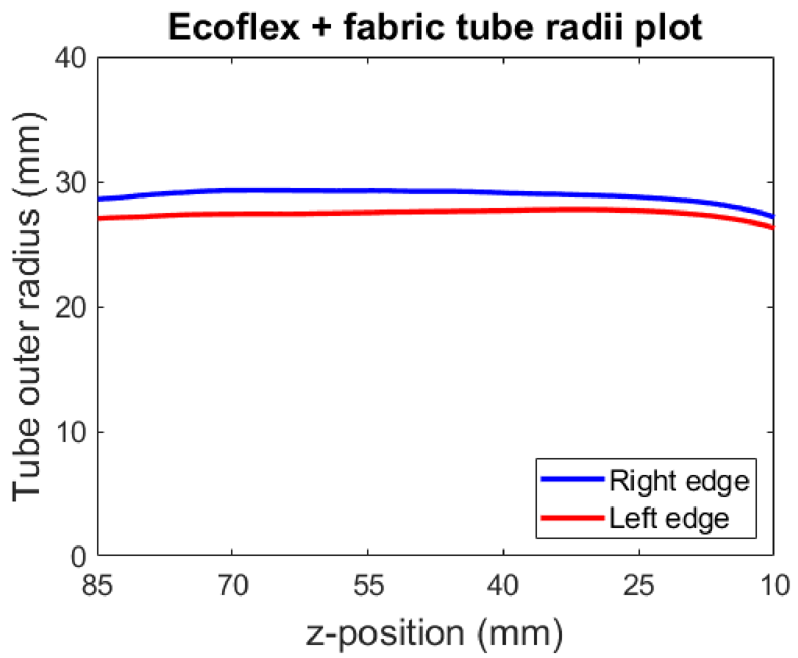

Figure S7: Radius measurement along the edges of a jacketed Ecoflex tube pressurized at 36.95 kPa, related to Figure 5.

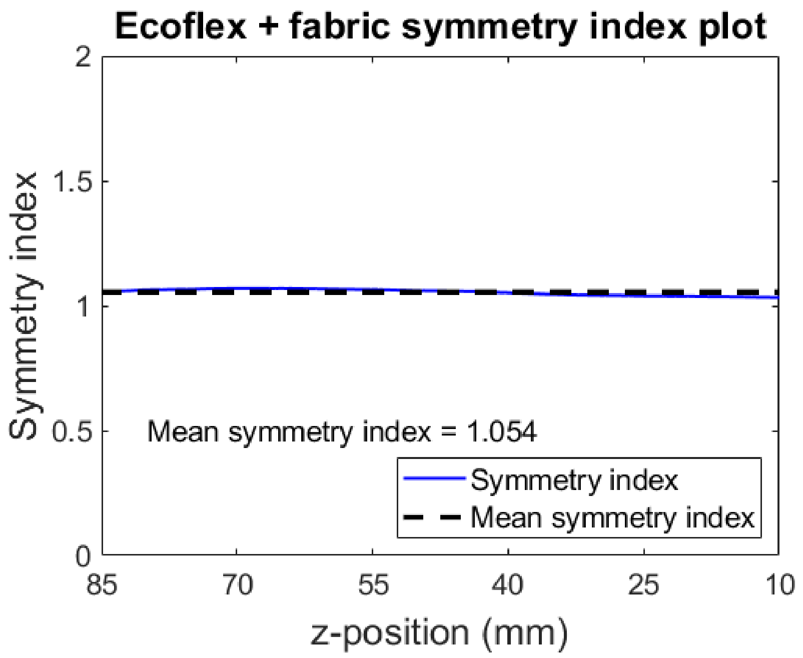

Figure S8: Symmetry index measurement of jacketed Ecoflex tube pressurized at 36.95 kPa, related to Figure 5.

SI - Constitutive Modeling of Elastomers and Fabrics

Elastomers (with or without fabric) are hyperelastic materials; this means their deformation behavior is governed by a strain energy density function instead of a single constant. The strain energy density W (J/mm^3) is a non-linear function of the principal strain invariants, which themselves are functions of the stretch ratios λ $(= 1 + \varepsilon)$ in the three principal directions.

Zhalmuratova *et al.* (Zhalmuratova, La, et al., 2019) performed a review of different constitutive hyperelastic material models to model the stress-strain behavior of fibre-elastomer composites. Of the models considered, the Mooney-Rivlin model (Rivlin, 1948) was used for elastomer strain energy density and the Holzapfel-Gasser-Ogden (HGO) model (Holzapfel, Gasser, and Ogden, 2000) was used for composite strain energy density. These are shown in Equations (S2) and (S3).

$$W_{elastomer} = C_{10}(I_1 - 3) + C_{01}(I_2 - 3) + \frac{1}{D_1 - 1}(J - 1)^2 \tag{S2}$$

$$W_{composite} = C_{10}(I_1 - 3) + \frac{k_1}{2k_2}\sum_{i=4,6}\left(\exp(k_2(I_i - 1)^2) - 1 \right) \tag{S3}$$

In these equations the variables C_{10}, C_{01}, D_1, k_1, and k_2 are all material constants and so must be experimentally determined, whereas J is the volume variation factor which, assuming the bulk incompressibility condition, can be assumed to be ≈ 1 (this eliminates the third term in Equation (S2)). I_1, I_2, I_4 and I_6 are the first, second, fourth, and sixth strain invariants, shown in Equations (S4)-(S7) (Bergstrom, 2015; Zhalmuratova, La, et al., 2019):

$$I_1 = \lambda_1^2 + \lambda_2^2 + \lambda_3^2 \tag{S4}$$

$$I_2 = \lambda_1^2\lambda_2^2 + \lambda_2^2\lambda_3^2 + \lambda_3^2\lambda_1^2 \tag{S5}$$

$$I_4 = \lambda_1^2 \cos^2\alpha + \lambda_2^2 \sin^2\alpha \tag{S6}$$

$$I_6 = (\lambda_1^4 \cos^2\alpha + \lambda_2^4 \sin^2\alpha)\cos\theta \tag{S7}$$

where α is defined as the angle between the fiber direction and stretching direction and θ is defined as the angle between the two fiber families (0 and 90 degrees respectively). This leads to Equations (S8) and (S9):

$$I_4 = \lambda_1^2 \tag{S8}$$

$$I_6 = 0 \tag{S9}$$

The strain energy density functions may be most easily converted to Cauchy stress equations under controlled conditions where the strain invariants are more easily determinable. For example, in a uniaxial tensile test where the sample is extended along axis 1, $\lambda_1 = \lambda_2^{-1/2} = \lambda_3^{-1/2}$. For a Mooney-Rivlin solid, the uniaxial stress is then described by Equation (S10) (Bergstrom, 2015):

$$t_{1,MR} = 2C_{10}(\lambda_1^2 - \lambda_1^{-1}) + 2C_{01}(\lambda_1 - \lambda_1^{-2}) \tag{S10}$$

Once the coefficients C_{10}, C_{01} have been fit to uniaxial test data, in theory they can be used to define the deformation behavior of the isotropic Mooney-Rivlin solid in any generalized stress state. This is the motivation behind the uniaxial testing conducted for this work.

The Holzapfel model is a decoupled strain energy density model for fiber-elastomer composites (Holzapfel, Gasser, and Ogden, 2000), where terms for strain energy density of the bulk elastomer and fabric layer can be considered separately, shown in Equation (S11):

$$W_{composite} = W_{elastomer} + \left(W_{fibres} + W_{interaction} \right) \tag{S11}$$

In the presented design in this work, the fabric is not embedded in the elastomer to prevent out-of-plane motion (as is the case with all true fiber-elastomer composites); therefore, it is assumed that $W_{interaction} \approx 0$. Meanwhile, it was also shown that $W_{elastomer}$ leads to the uniaxial stress formula in Equation (S10) (the C_{01} term is omitted in the simulation due to a limited impact on the deformation behavior at higher strains). Therefore, combining Equation (S3) with Equation (S11) leads the following equation for the strain energy density of the fabric:

$$W_{fabric} = W_{fibres} = \frac{k_1}{2k_2} \sum_{i=4,6} \left(\exp(k_2(I_i - 1)^2) - 1 \right) \tag{S12}$$

which, combined with Equations (S8) and (S9), leads to Equation (S13) for the uniaxial tensile stress of fabric.

$$t_{1,fabric} = 2k_1\lambda_1(\lambda_1^2 - 1)\exp(k_2(\lambda_1^2 - 1)^2) \tag{S13}$$

SI - Hysteresis Testing

Additional information about the elastomer resins from the Smooth-On, Inc.^{TM} website is provided in Table S2 (Smooth-On, Inc., n.d.[a]; Smooth-On, Inc., n.d.[b]).

Table S2: Additional material properties of elastomers. Data obtained from manufacturer website. Related to STAR Methods.

**Property**	**Ecoflex 00-50**	**Dragon Skin 10 SLOW**
Tensile Strength (psi)	315	475
Shore Hardness	00-50	10A
Specific Gravity (g cm^{-3})	1.07	1.07
Elongation at Break (%)	980	1000
Mixed Viscosity (cps)	8000	23000

Complete quasi-hysteresis plots for all materials are shown. The legend associates the colour of each plot curve with the maximum level of strain for that test. With the exception of some of the last entries in the legend, there are two curves for each colour: one with higher stress values and one with lower. These correspond to the first and third tensile tests to that level of strain, respectively.

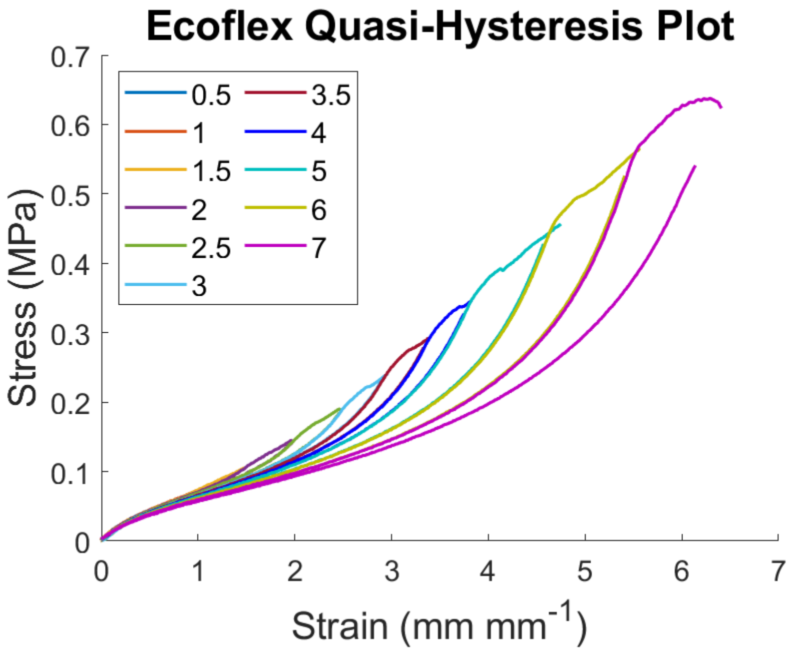

Figure S9: Ecoflex quasi-hysteresis plot, related to Figure 3.

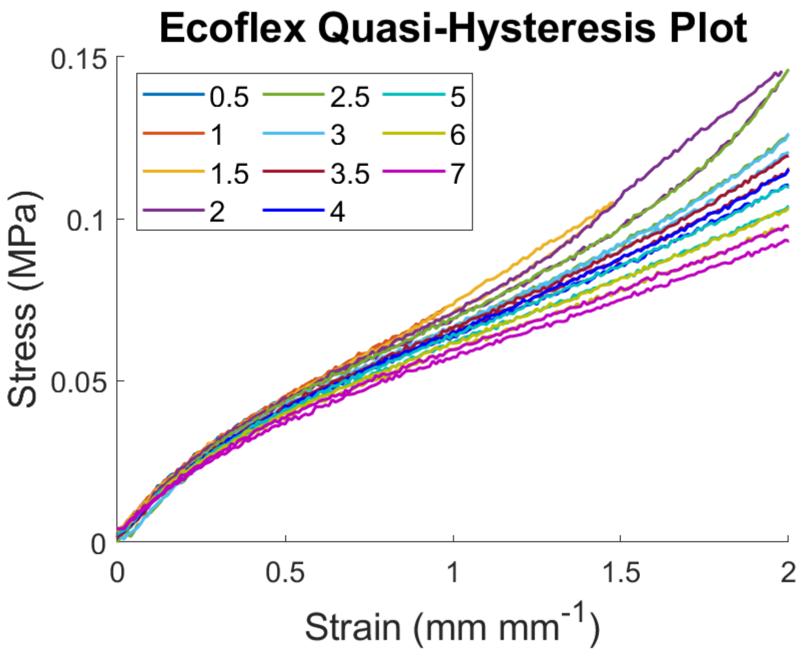

Figure S10: Ecoflex quasi-hysteresis plot (closeup), related to Figure 3.

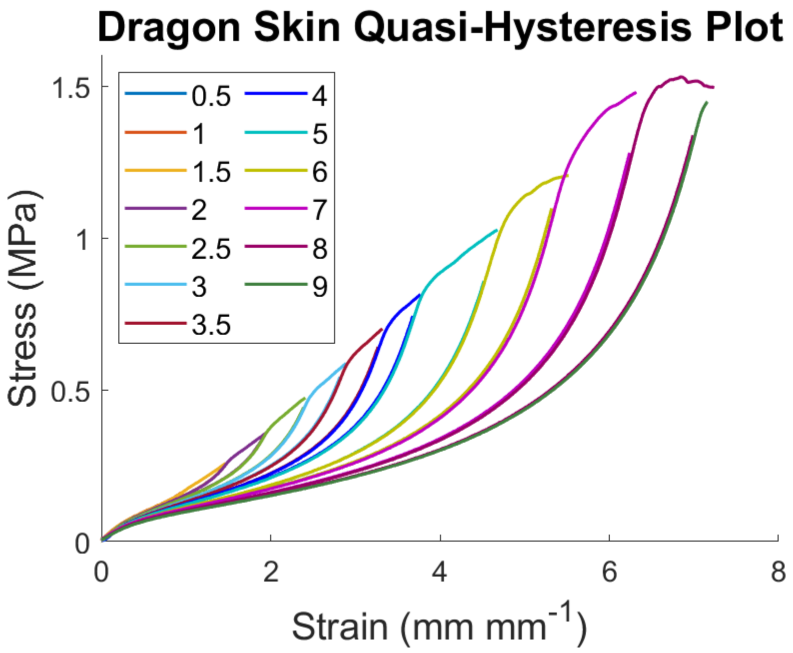

Figure S11: Dragon Skin quasi-hysteresis plot, related to Figure 3.

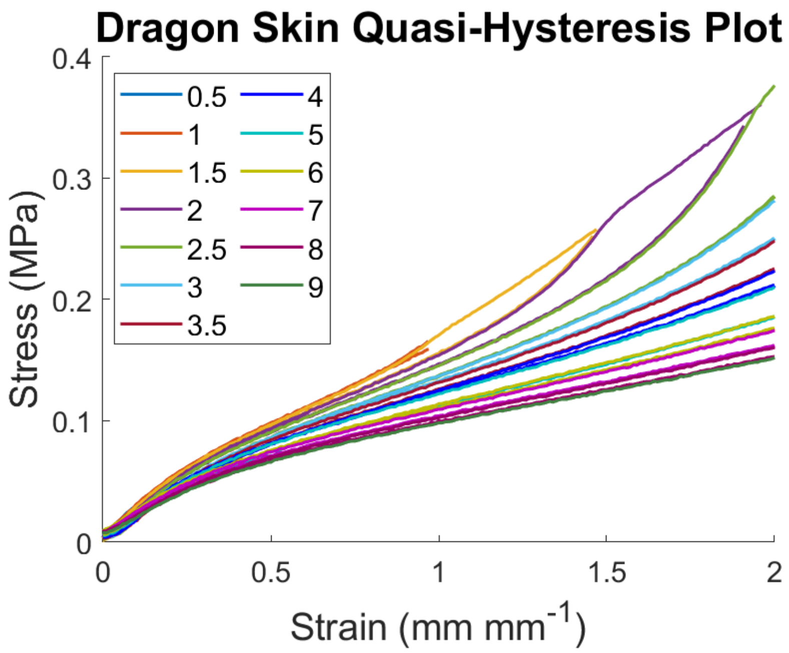

Figure S12: Dragon Skin quasi-hysteresis plot (closeup), related to Figure 3.

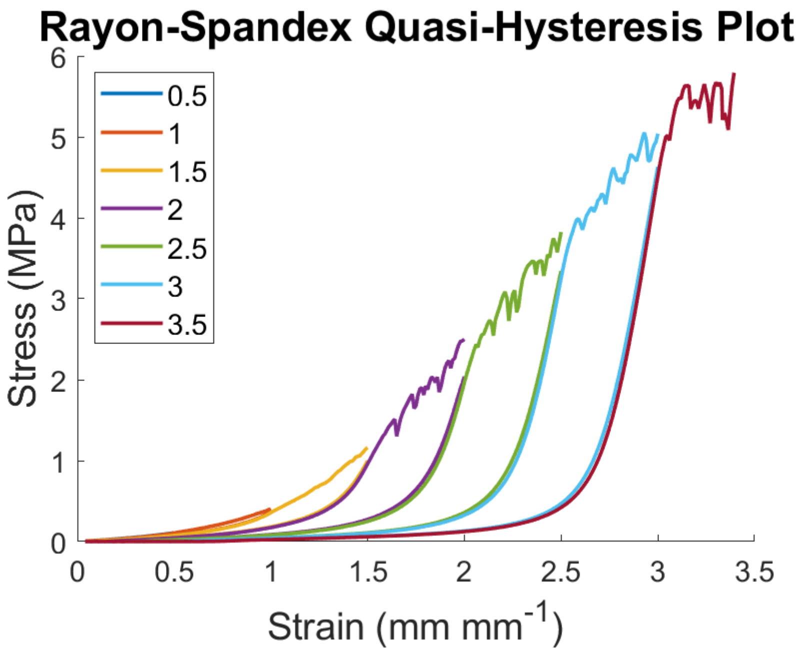

Figure S13: Rayon-spandex fabric quasi-hysteresis plot, related to Figure 3.

It was asserted in the main text that fitting the constitutive model coefficients to a selected curve from the quasi-hysteresis plot was a necessity in order to not overestimate the stiffness of the material; a simulation-based justification for this reasoning is provided here. In Figure S14a, theoretical stress-strain curves were obtained by fitting coefficients to the uniaxial

stress-strain curve of virgin Ecoflex and the selected curve from the quasi-hysteresis test. Then, finite element models were designed in which the material behavior was defined using each set of coefficients, and each was pressurized to 13.99 kPa of hydrostatic pressure. As shown in Figure S14b, the stretched tube distended 41% more than the virgin tube.

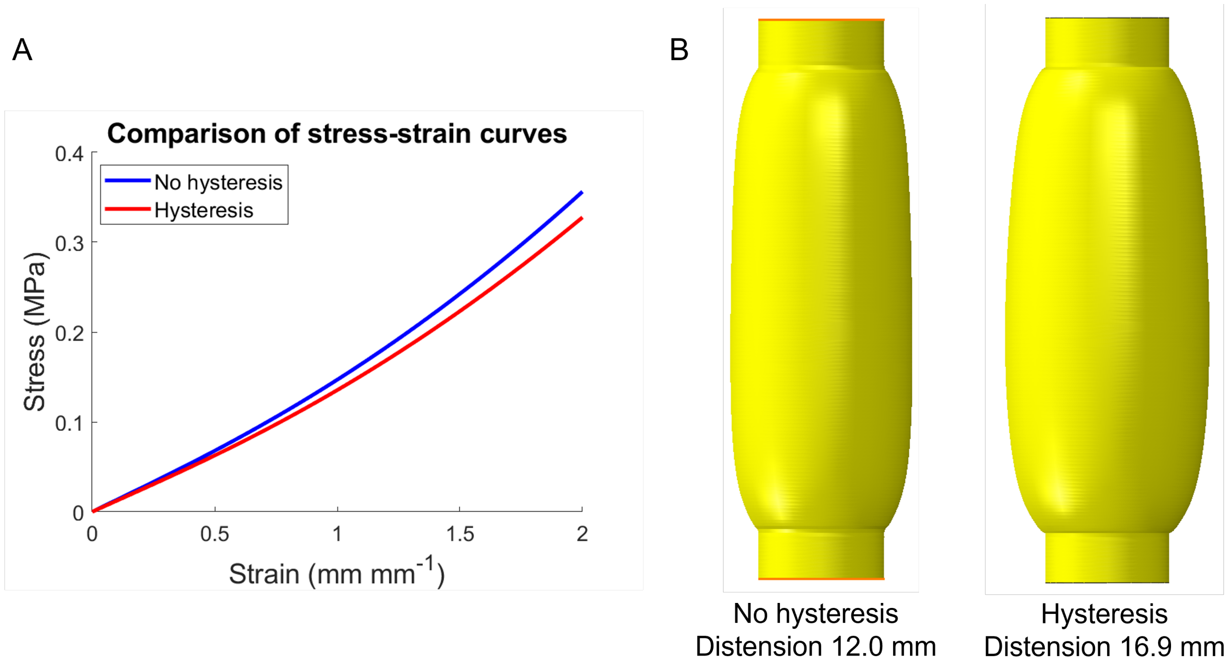

Figure S14: Constitutive model coefficients cannot be fit to the stress-strain curve of the virgin material or they will overestimate the stiffness. (A) Theoretical stress-strain curves obtained from coefficients fitted to uniaxial stress-strain curve of virgin material (blue) and the selected quasi-hysteresis curve (3rd test to 200% strain; red). (B) Finite element models of Ecoflex tubes at 13.99 kPa hydrostatic pressure, defined using virgin material coefficients (left) and stretched material coefficients (right). Related to Figure 3.

Plots of the theoretical stress-strain curves using the fitted coefficients for all materials, overlaid with the selected quasi-hysteresis curves (3rd test to 200% strain), are shown below. Coefficients for elastomers were selected by fitting to the first half of the the selected quasi-hysteresis curve (up to 100% strain), while coefficients for the fabric were selected by fitting to the entire selected quasi-hysteresis curve.

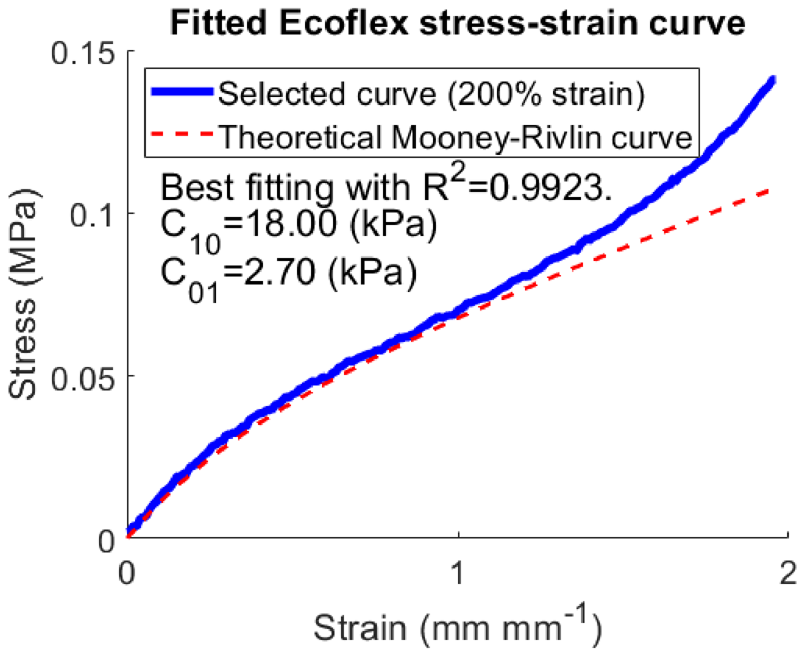

Figure S15: Theoretical stress-strain data using fitted Ecoflex coefficients, related to Figure 3.

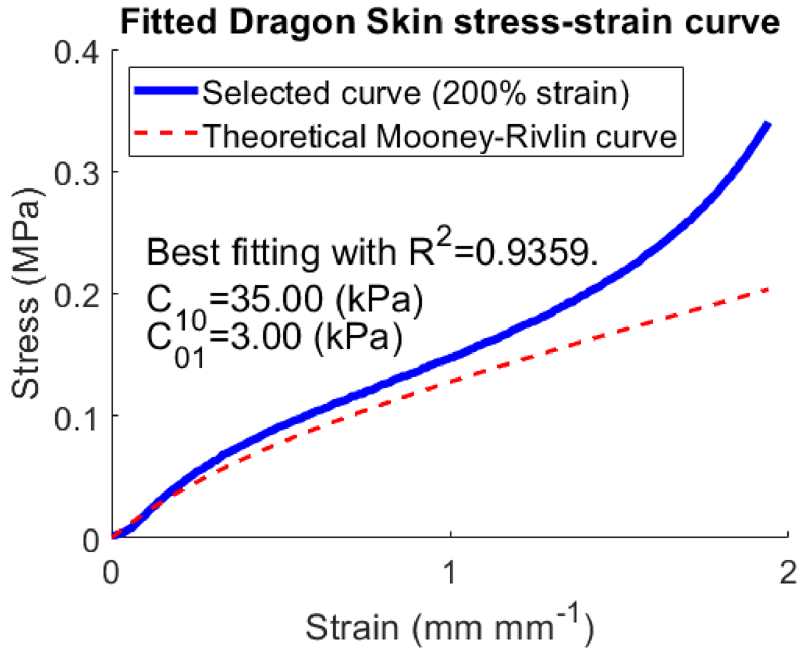

Figure S16: Theoretical stress-strain data using fitted DragonSkin coefficients, related to Figure 3.

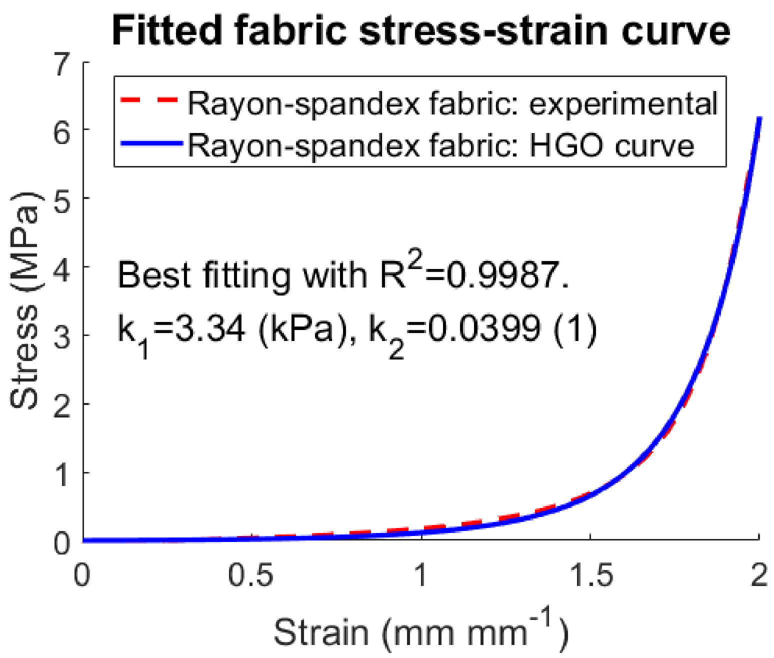

Figure S17: Theoretical stress-strain data using fitted rayon-spandex fabric coefficients, related to Figure 3.

Finally, plots of the theoretical stress-strain curves for the elastomer and fabric combined, using the fitted coefficients, are shown below.

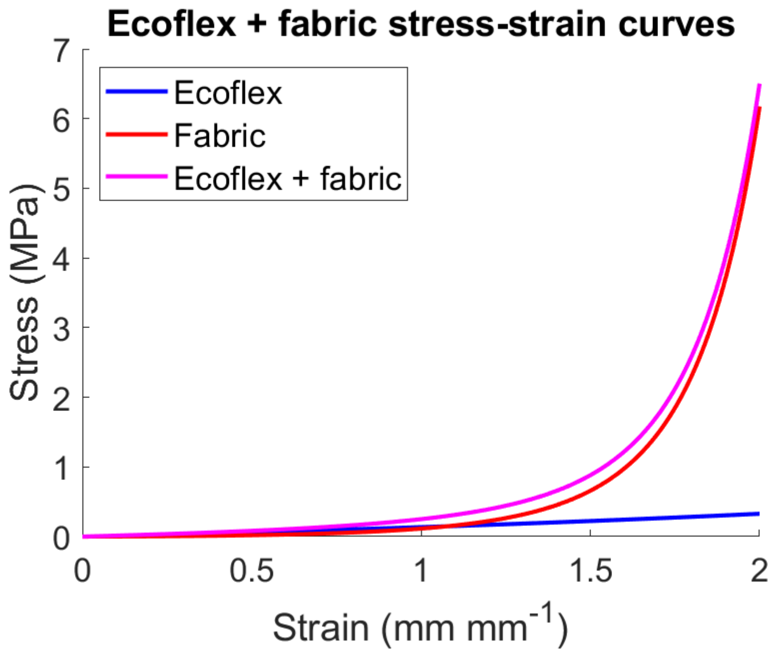

Figure S18: Theoretical stress-strain curve for Ecoflex and fabric, related to Figure 3.

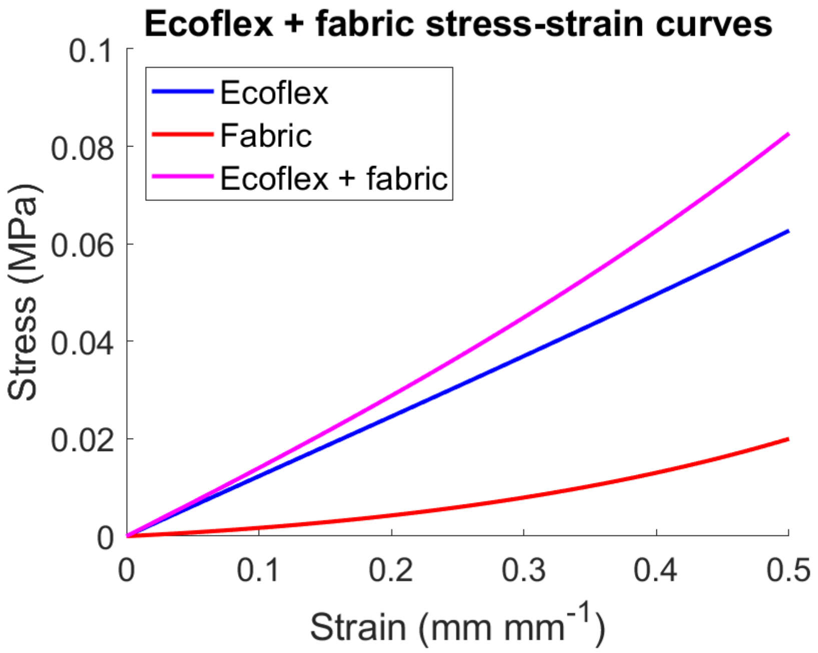

Figure S19: Theoretical stress-strain curve for Ecoflex and fabric (closeup), related to Figure 3.

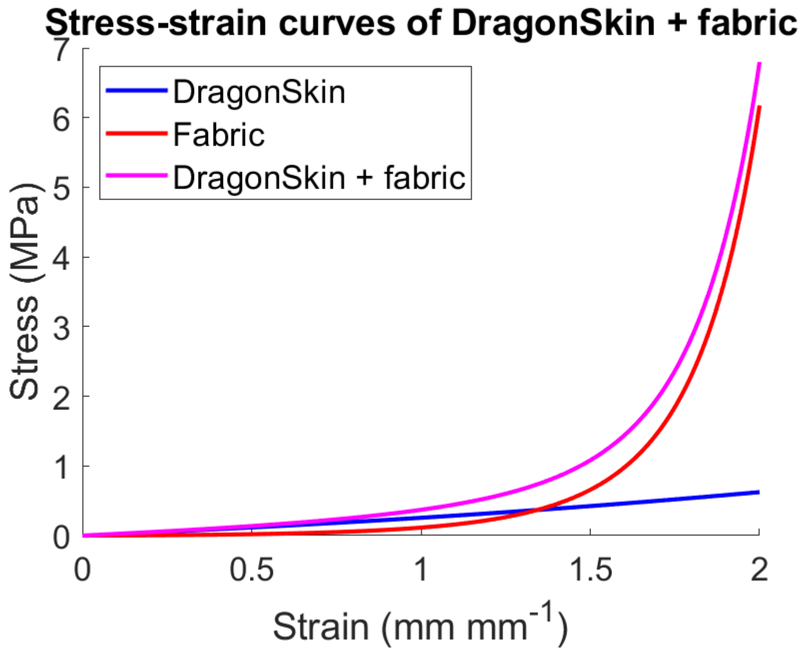

Figure S20: Theoretical stress-strain curve for Dragon Skin and fabric, related to Figure 3.

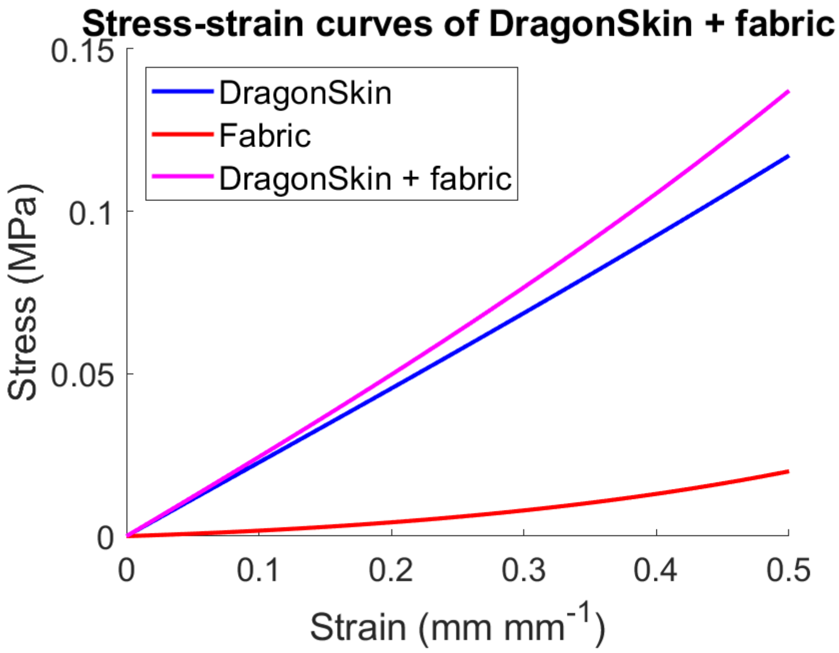

Figure S21: Theoretical stress-strain curve for DragonSkin and fabric (closeup), related to Figure 3.

SI - Finite Element Simulations

Prior to analyzing the history outputs, the ABAQUS simulations can be stress- and strain-mapped to achieve a qualitative (and roughly quantitative) preliminary analysis of the deformed configuration. An example is shown in Figure S22.

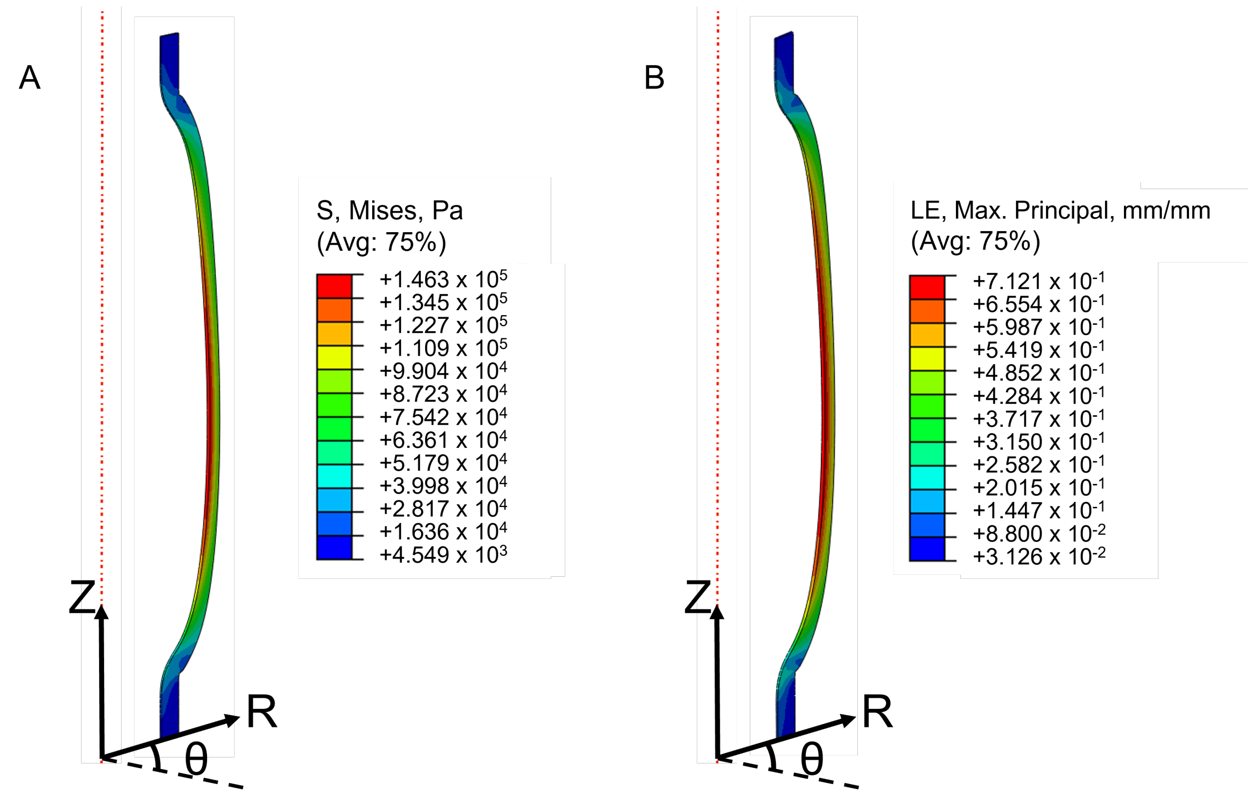

Figure S22: Color-mapped ABAQUS simulations of an Ecoflex tube distended under 13.99 kPa of hydrostatic pressure. (A) Color mapping of von Mises stress on deformed tube simulation. (B) Color mapping of logarithmic strain magnitude on deformed tube simulation. Related to Figure 4.

Plots of the simulation data for tube distension, rate of distension, tube volume, and rate of volumetric expansion are shown below.

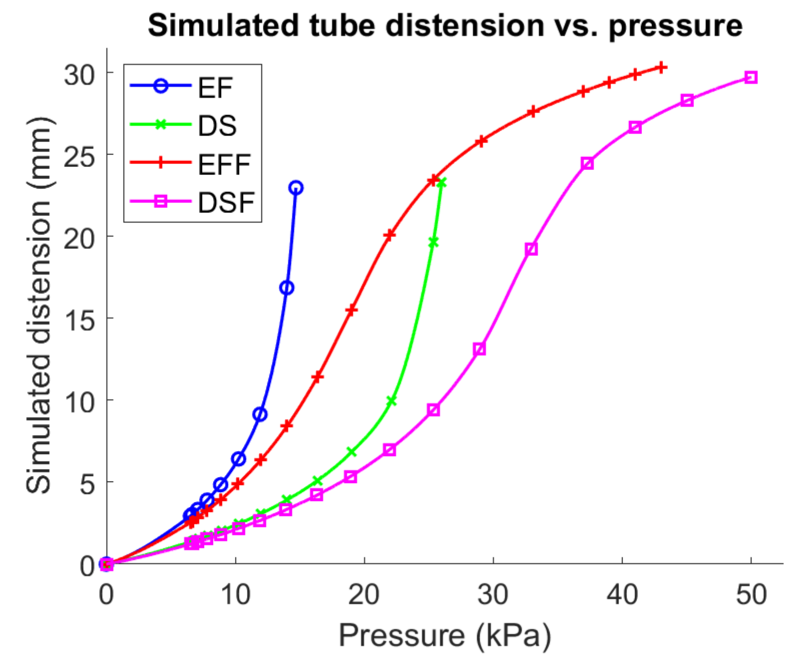

Figure S23: Simulated distension vs. pressure plot, related to Figure 4.

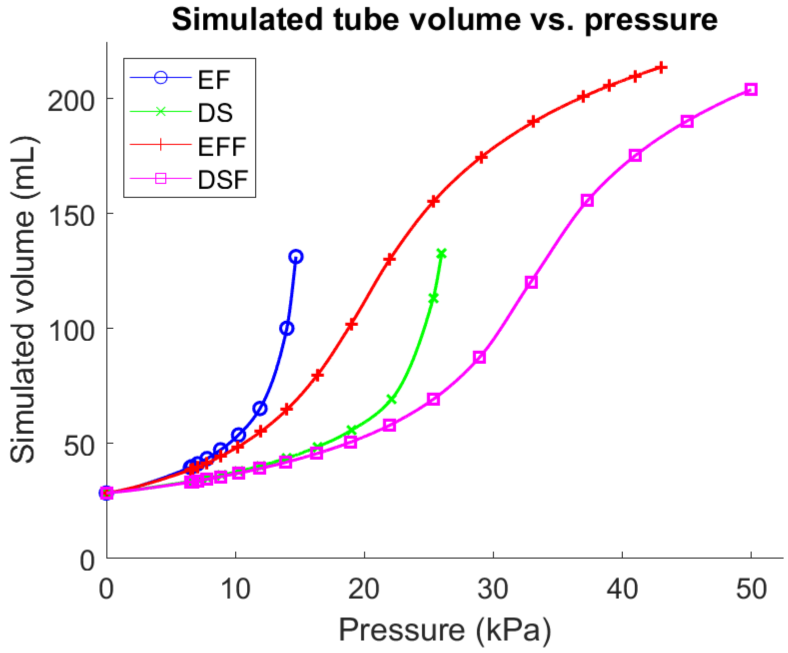

Figure S24: Simulated volume vs. pressure plot, related to Figure 4.

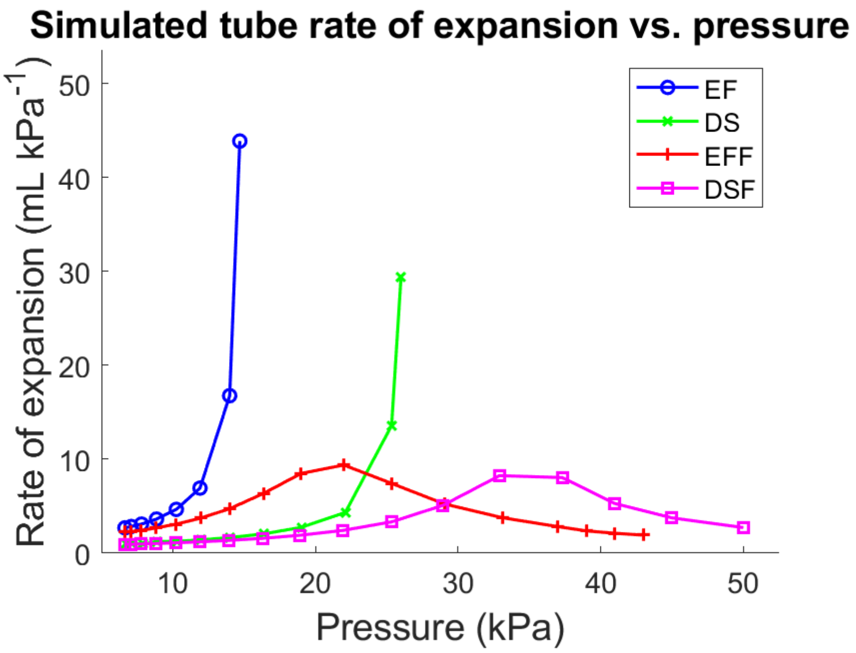

Figure S25: Simulated rate of distension vs. pressure plot, related to Figure 4.

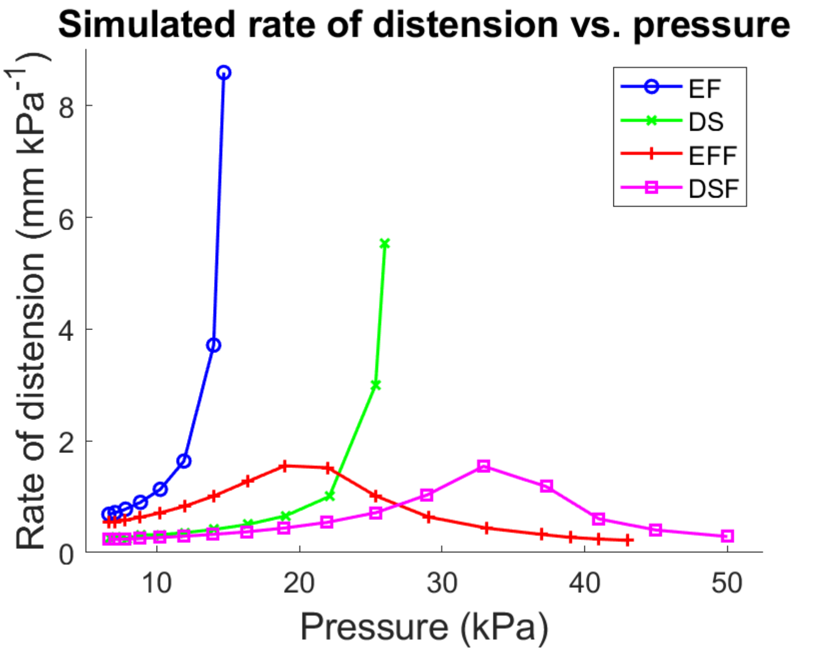

Figure S26: Simulated rate of expansion vs. pressure plot, related to Figure 4.

SI - Comparison of Experiment and Simulation Results

Comparison plots of tube distension, rate of tube distension per unit pressure increase, tube volume, and rate of volumetric expansion per unit pressure increase are shown in Figures S27-S30. Superimposition of the experimental and simulated data allows for visual confirmation of the agreement between the two sets.

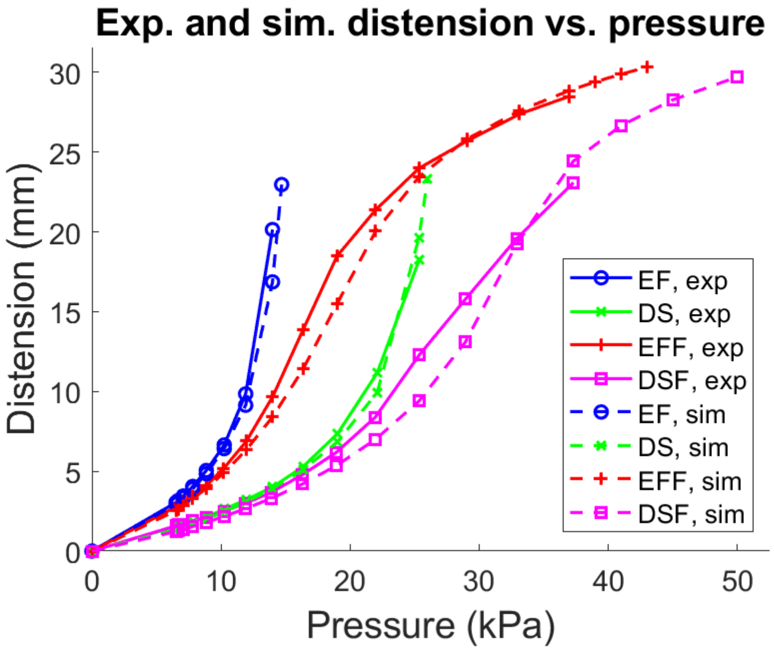

Figure S27: Comparison of experimental and simulated distension values, related to Figure 4.

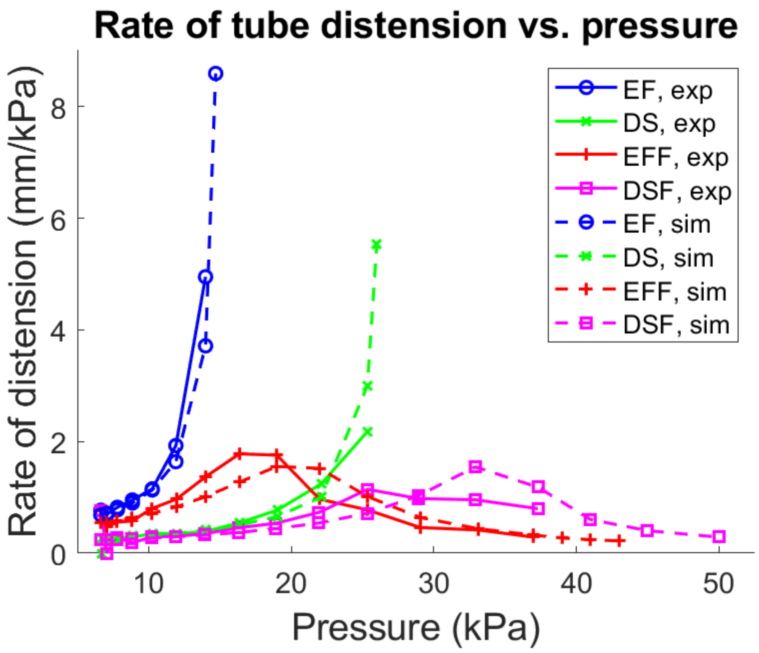

Figure S28: Comparison of experimental and simulated rate of distension values, related to Figure 4.

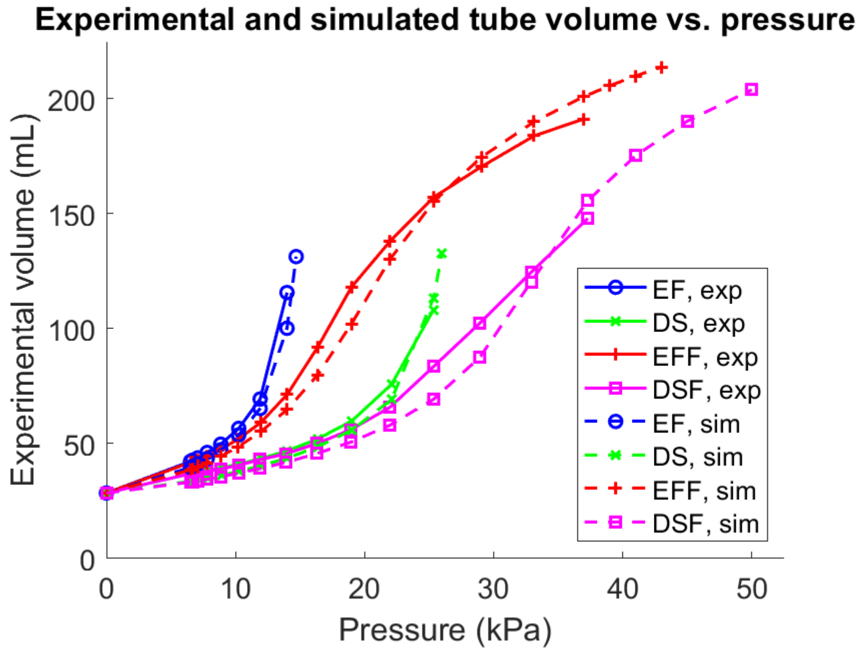

Figure S29: Comparison of experimental and simulated volume values, related to Figure 4.

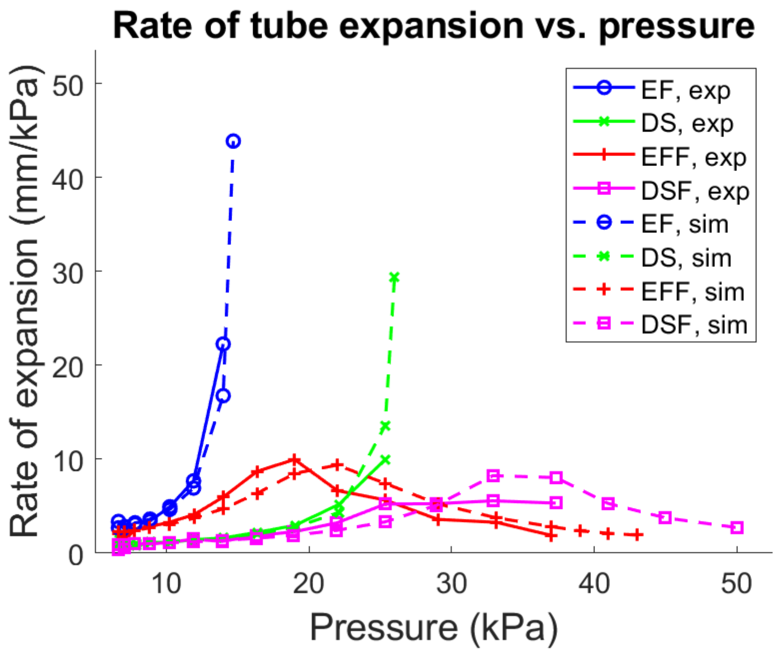

Figure S30: Comparison of experimental and simulated rate of expansion values, related to Figure 4.

Alternatively, least-squares regression parity plots to quantitatively illustrate the similarity between the experimental and simulated data sets are below in Figures S31-S38. All R^2 values are > 0.9, indicating high correlation between the sets.

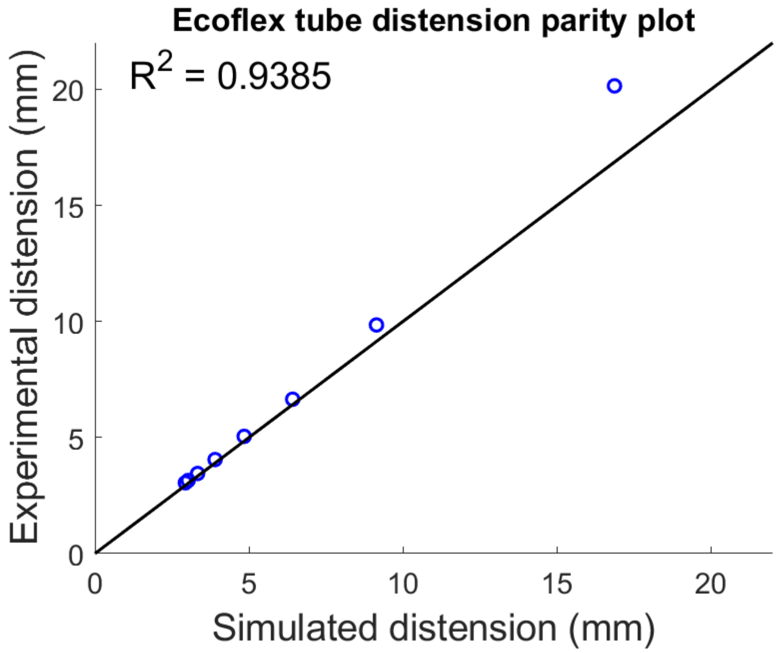

Figure S31: Ecoflex tube distension parity plot, related to Figure 4.

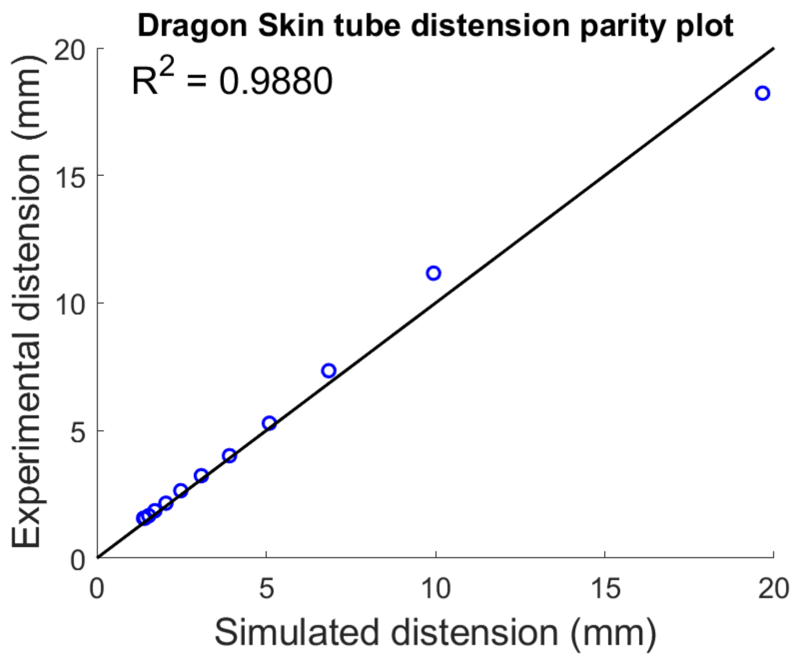

Figure S32: Dragon Skin tube distension parity plot, related to Figure 4.

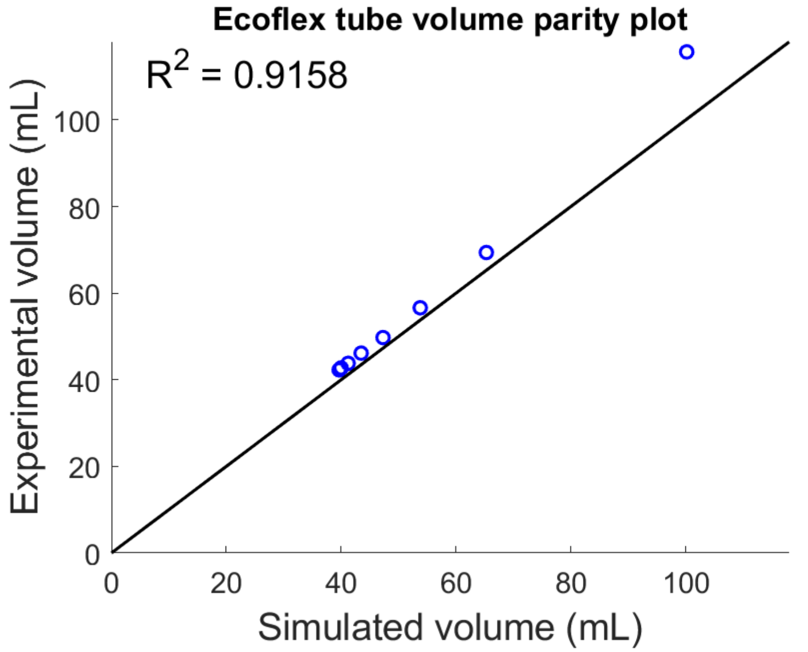

Figure S33: Ecoflex tube volume parity plot, related to Figure 4.

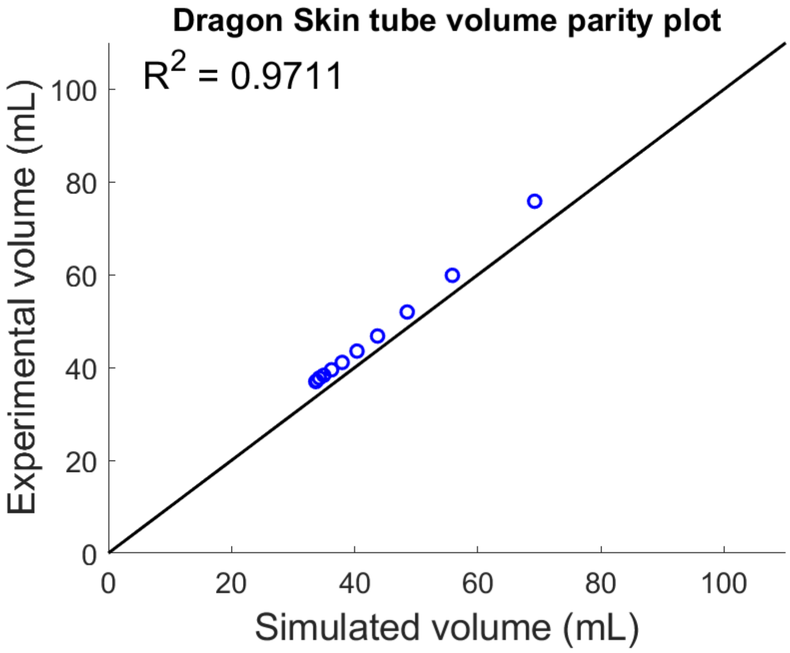

Figure S34: Dragon Skin tube volume parity plot, related to Figure 4.

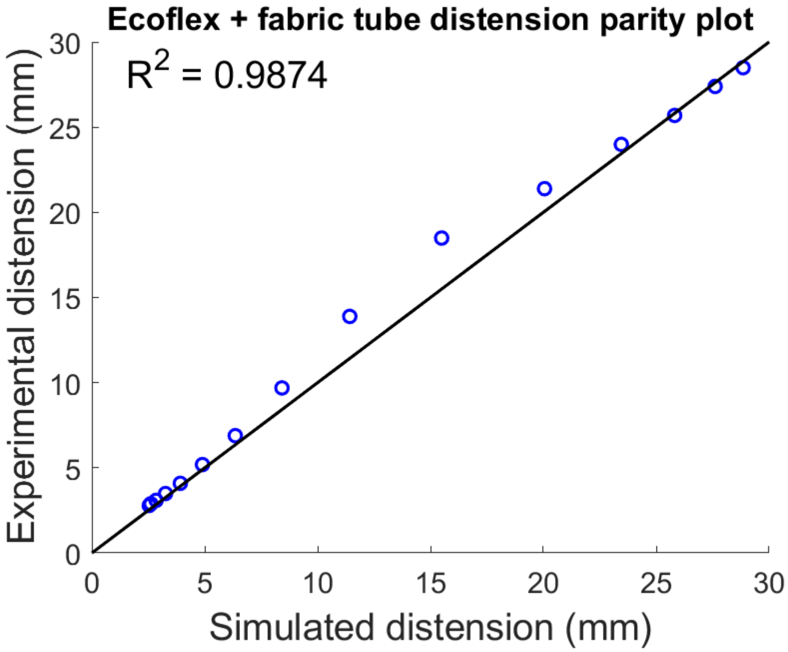

Figure S35: Ecoflex + fabric tube distension parity plot, related to Figure 4.

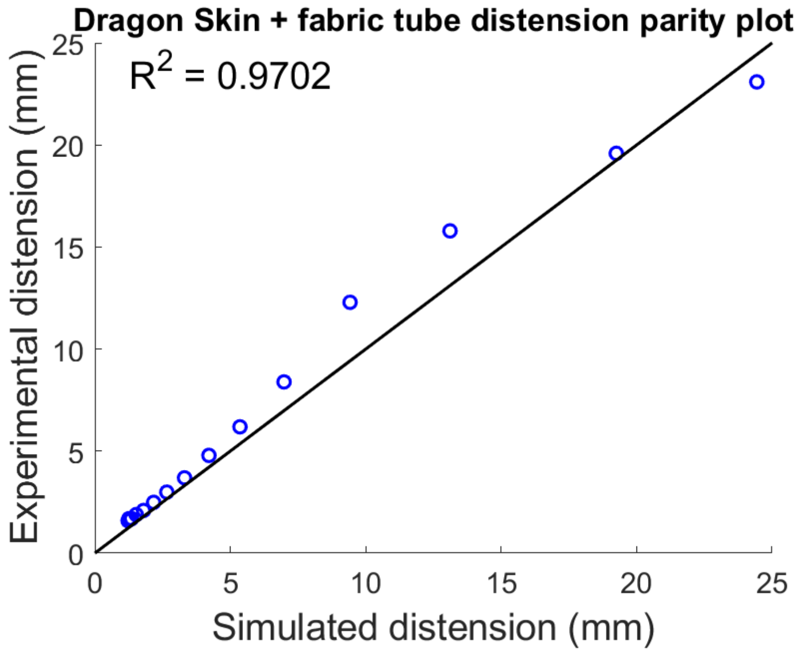

Figure S36: Dragon Skin + fabric tube distension parity plot, related to Figure 4.

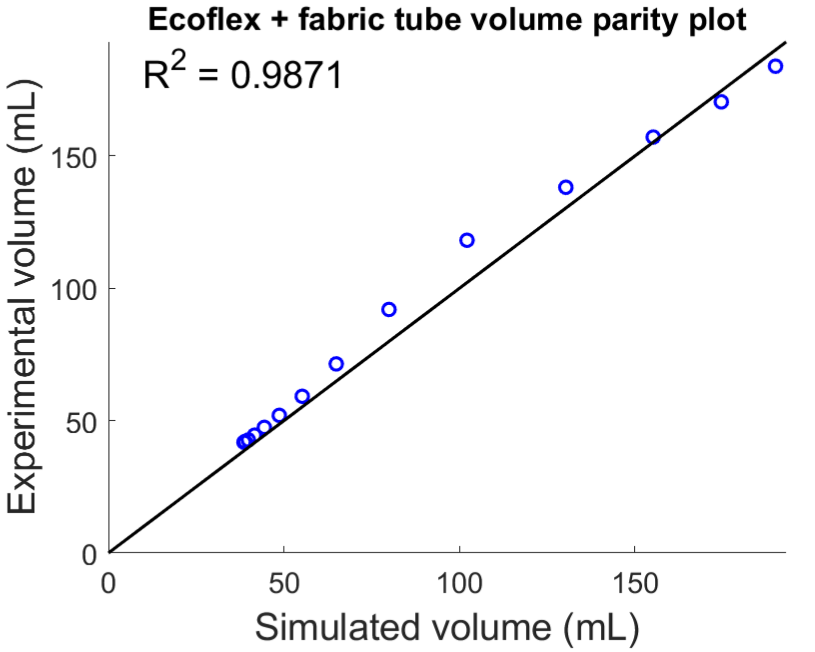

Figure S37: Ecoflex + fabric tube volume parity plot, related to Figure 4.

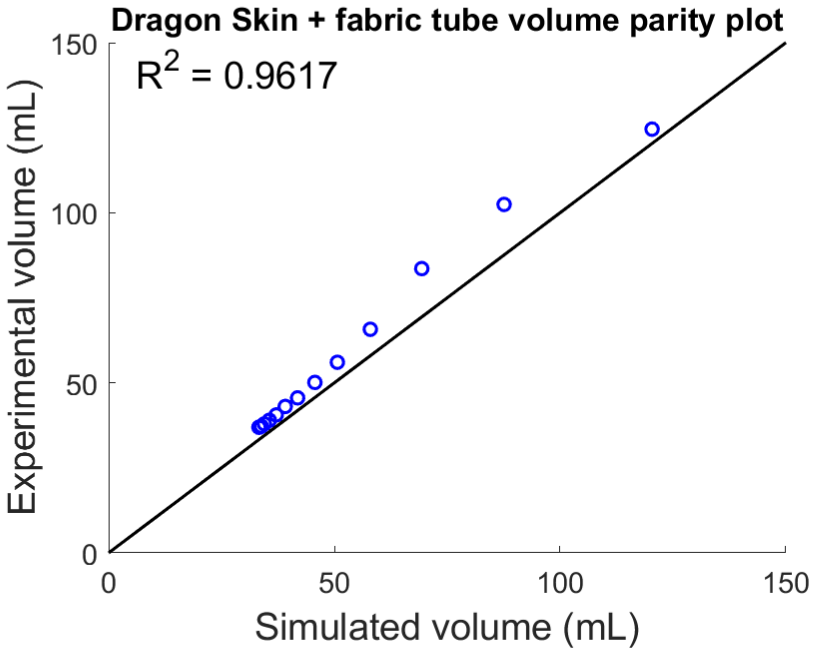

Figure S38: Dragon Skin + fabric tube volume parity plot, related to Figure 4.
